# Canine distemper virus phylogenetic structure and ecological correlates of infection in mesocarnivores across anthropogenic land use gradients

**DOI:** 10.1128/spectrum.01225-24

**Published:** 2025-03-03

**Authors:** Jonathan Wilson, Samantha Rubio, Liliana C. M. Salvador, Nicole M. Nemeth, Jillian D. Fishburn, Nicole L. Gottdenker

**Affiliations:** 1 Department of Pathology, College of Veterinary Medicine, University of Georgia 70734 , Athens, Georgia, USA; 2 School of Animal and Comparative Biomedical Sciences, The University of Arizona 8041 , Tucson, Arizona, USA; 3 Southeastern Cooperative Wildlife Disease Study, College of Veterinary Medicine, University of Georgia 70734 , Athens, Georgia, USA; 4 Athens Veterinary Diagnostic Laboratory, College of Veterinary Medicine, University of Georgia 70734 , Athens, Georgia, USA; Changchun Veterinary Research Institute, Chinese Academy of Agricultural Sciences, Changchun, China

**Keywords:** canine distemper virus, *Morbillivirus*, mesocarnivores, anthropogenic land use, phylogenetic structure, disease ecology, raccoon, gray fox, striped skunk

## Abstract

Anthropogenic land use impacts infectious diseases at the wildlife-domestic-human interface by changing host spatial distribution, behavior, density, and population dynamics. Canine distemper virus (CDV) is a significant cause of morbidity and mortality in many wild and domestic animals. Given the propensity of CDV to infect synanthropic mesocarnivores, it is important to investigate host and environmental factors affecting mesocarnivore CDV infection. Here, we investigated patterns of CDV infection and developed a statistical model to identify environmental variables related to CDV risk in commonly affected mesocarnivores. We sampled carcasses (*N* = 270) submitted to the Southeastern Cooperative Wildlife Disease Study from January 2019 to December 2022 and sequenced the CDV H-gene of 32 CDV-positive animals. Overall, 158 out of 270 mesocarnivores (58.5%) and four species (raccoon, red fox, gray fox, and striped skunk) were diagnosed with CDV across 13 states. Ripley’s K analysis showed positive cases were more spatially clustered at larger distances than expected due to chance. A generalized linear model for CDV-infected animals showed surface imperviousness, precipitation, and subadult/adult age classes were significant positive explanatory variables, but elevation had a significant negative association with CDV infection likelihood. H-gene sequence diversity among wild mesocarnivores in the southeastern United States was geographically separated into groups east and west of the Mississippi River, with only two eastern samples clustering with western groups. By identifying areas of intense human development at the highest risk for CDV, it may be possible to focus surveillance efforts in these areas, allowing for earlier outbreak identification, potentially preventing cross-species CDV transmission.

**IMPORTANCE:**

Anthropogenic land use change can impact infectious disease spread by altering animal distribution and behavior. Canine distemper virus (CDV) is a significant cause of morbidity and mortality in wild and domestic carnivores. This study investigated how land use influences CDV infection in wild carnivores by examining tissues collected between 2019 and 2022 from wild carnivores found dead in the southeastern United States. CDV strains were geographically distinct, with differences between populations east and west of the Mississippi river. Statistical models showed areas with increased human development and higher precipitation had higher CDV risk; however, there was lower risk associated with higher elevations and younger animals. The importance of this study is that it identifies geographic structure of CDV in the southern United States, and identifies land-use associations with potential high-risk areas for CDV transmission-information that is useful for wildlife disease surveillance and control strategies.

## INTRODUCTION

Anthropogenic land use has a significant impact on the population structure and dynamics of infectious diseases at the wildlife-domestic-human interface (1–3). Proposed mechanisms by which anthropogenic land use (e.g., deforestation, silviculture, agricultural activities, urbanization, and suburbanization) can alter the structure and spread of infectious disease within and between wildlife populations, domestic animals, and humans include altered host density, behavioral changes (including contact within and between species and movement of hosts), and impaired host immune function (4–7) .

In most cases of directly transmitted pathogens, host density drives contact rates and thus pathogen spread (8). High densities usually result in increased intra and interspecific contact within and between species and individuals and subsequently cause a greater prevalence of infection (9, 10). This idea of higher density populations and greater contact rates leading to more disease is of particular importance in synanthropic species that thrive in human-disturbed environments where supplemental resource availability, shelter, and lack of predation can harbor large populations (11–13). Despite this, some studies have suggested that highly urbanized areas may dampen the spread of infectious diseases among wildlife due to reduced population sizes (14). Thus, it remains unclear how continued urban development affects the dynamics of directly transmitted pathogens in synanthropic species. Therefore, it is crucial to understand the relationships among land use change, the role of human development, and infectious disease transmission in synanthropic wildlife to develop effective management and conservation strategies to mitigate the negative impacts of disease and protect affected species and their ecosystems.

Canine distemper virus (CDV) belongs to the genus *Morbillivirus*, family Paramyxoviridae, and represents a valuable study system for investigating land use-infectious disease relationships in multi-host (wildlife and domestic animal) systems. CDV infects a wide variety of carnivore hosts and rarely other species groups (15, 16). CDV has also been implicated in severe population declines in multiple species, including the near-extinction of the black-footed ferret (*Mustela nigripes*) in the United States (US) (17). CDV causes an important disease in domestic dogs and can be transmitted between wildlife and dogs and vice versa (18). CDV has also been proposed as a risk to human health, and it is hypothesized that waning population-level measles immunity may leave humans susceptible to CDV infection (19, 20). CDV infection is commonly reported in the raccoon (*Procyon lotor*) (21–24), a synanthropic mesocarnivore that lives in high density and comprises well-connected populations in urbanized areas in North America (25, 26). However, there is an incomplete understanding of CDV infection dynamics within many multi-host systems, such as carnivore communities. The role that different carnivore species play in the maintenance and spread of CDV is not understood and consequently, strategies to target mitigation measures are not well informed.

The southeastern US has a wide variety of potential CDV host species. Preliminary work from postmortem diagnostic data of CDV-infected wild carnivores demonstrated that CDV is widespread, with at least nine carnivore species across southeastern states experiencing mortality attributed to CDV infection. In the most commonly infected species, the raccoon and gray fox (*Urocyon cinereoargenteus*), there appears to be a trend of cases clustering in suburban areas with fewer cases occurring in highly urbanized and rural areas (27). Studies in other parts of the world have suggested that the dynamics of CDV outbreaks can vary over time and space (28). Given the propensity of CDV to infect synanthropic mesocarnivores, it is important to investigate whether there are human land use features that affect the likelihood of virus transmission among wildlife.

Here, we investigated the phylogenetic structure and spatial patterns of CDV infection in wild mesocarnivore carcass submissions from 13 states from January 2019 to December 2022. The objectives of this study were to explore patterns of CDV genetic diversity in wild mesocarnivores submitted from 13 southeastern states, investigate the spatial distribution of CDV in free-ranging mesocarnivores from the same region from 2019 to 2022, and identify ecological factors that may increase the risk of CDV. Specifically, we aimed to investigate how land use influences the likelihood of severe, fatal CDV infection (i.e., diagnosed postmortem) in wild mesocarnivores.

## MATERIALS AND METHODS

The Southeastern Cooperative Wildlife Disease Study (SCWDS, University of Georgia; Athens, Georgia, USA) incorporates 17 wildlife agency member states, most of which are in the southeastern US. For this project, we used a data set consisting of diagnostic submissions of all mesocarnivores (*N* = 270) submitted to SCWDS from January 2019 to December 2022 (opportunistic sampling). The raw data from SCWDS included the variables: state, county, area, coordinates where animal was found (when provided), species, date, sex, age, weight, and diagnoses. Additionally, the land cover data for each location were extracted from raster maps available from the National Land Cover Database (NLCD). The different land cover types are described in Table S1. The classification system used by NLCD is modified from the Anderson Land Cover Classification System (29). Along with elevation data from the *elevatr* package (30), average temperature and precipitation values were accessed from the PRISM database (31). Further variables calculated for each data point were distance to the nearest hydrological feature and distance to the nearest other distemper case in the data. The hydrological maps were accessed from the TIGER database (32).

The submitted wild animals were either found dead or moribund and were subsequently euthanized. If the animal was observed alive, clinical signs were often described and included lethargy, lack of fear of humans, remaining in the same place for a day or more, visible during daylight hours, and sometimes neurological signs (e.g., mental dullness, confusion, ataxia, and disorientation). All submissions (*N* = 270) were evaluated for CDV infection at necropsy by histopathology, and/or CDV antigen detection by direct fluorescent antibody (DFA) testing of fresh or frozen brain samples (33), and/or immunohistochemistry (IHC) for CDV (34). Histopathology findings attributable to CDV included depletion and necrosis of lymphatic tissue, nonsuppurative interstitial pneumonia and/or respiratory epithelial hyperplasia, inflammation with accumulation of inflammatory cells in the lumen of the airways with or without viral syncytia and/or intranuclear and intracytoplasmic inclusion bodies in respiratory, urinary, and/or gastrointestinal epithelium. Nervous system histopathology associated with CDV included neuronal degeneration, gliosis, demyelination, perivascular cuffing, leptomeningitis, and/or inclusion bodies in the cytoplasm of neurons and glial cells. Gastrointestinal lesions, when present, include inflammation, necrosis, and the presence of lymphocytes and macrophages in the lumen. CDV lesions vary depending on the stage of the disease and affected organ and may include mild inflammation in early stages, and severe inflammation and necrosis in later stages (35, 36). Final diagnoses of submitted cases testing negative for CDV (negative FA and no CDV-pathognomonic lesions on histopathology with negative IHC for animals where lesions included CDV in their differential histopathologic diagnosis) were assumed to become ill or died from causes not related to CDV.

### Statistical analysis of submitted cases

Diagnostic data from SCWDS cases of CDV from January 2019 to December 2022 were imported into R (version 4.2.0.) (37). All analyses described below were conducted in the R programming environment. A logistic link function was applied, and a binomial error distribution was assumed. A positive or negative diagnosis of canine distemper in wild mammal necropsy case submissions was the response variable, whereas species, location, sex, age, month received, distance to nearest case (identified in the model as knn.dist), elevation, precipitation, temperature, distance to water source, surface imperviousness, and land cover type were explanatory factors. The model was fitted using maximum likelihood estimation. To select the best model, different models were fitted and compared based on their Akaike information criterion (AIC) (38). The general workflow involved creating a global model involving all the variables at a basic level. The “add1()” function from the *stats* package was then used to assess interacting variables. Interacting variables that made a significant improvement to the AIC were added to the model. Next, the “dropterm()” function from the *MASS* package (39) was used to evaluate the impact of removing each variable from the model using AIC. Those variables that had a negative influence on AIC were removed from the model. The functions “influencePlot(),” and “outlierTest()” from the *car* package (40) were used to evaluate for outliers in the data that may have had a significant impact on the model fit. AIC was again used to evaluate the model improvements. Three outliers were removed from the data that had a significant influence on the model via the influence plots and outlier test because it was determined that they were data entry errors that could not be retrieved/corrected. The final model underwent testing to ensure proper data fit by plotting Pearson’s residuals against fitted values. The data set was initially split into a training and test set. The predictive ability of the model was then evaluated on the test set. Splitting a data set into training and test sets is a model validation approach that helps avoid overfitting and underfitting, minimizes bias in the model evaluations, and assesses model performance on unseen (test) data (41). Finally, the model coefficients and their standard errors were interpreted to understand the relationships between the predictor variables and the response variable. The significance of the predictor variables was determined using *P* values. Forest plots of standardized residual predictors of the best-fit model were also created (Fig.S3). Additional diagnostics (influence plots, residuals vs fitted values, Normal *Q*-*Q* plots of theoretical vs sample quantiles were performed on the best-fit model) (Fig.S1) and DHARMa residual diagnostics to evaluate model performance were also plotted (Fig. S2) (42). Marginal effects plots were used to evaluate the relationships between best-fit model significant predictor variables and interactions and the probability of CDV infection using the ggeffects package (43).

### Nucleic acid detection/sequencing/analysis

Tissue samples (most often brain, but also lung, liver, and spleen) were collected from a subset of 32 known CDV-positive cases for viral RNA extraction. CDV RNA was extracted from tissue samples with a commercially available extraction kit (RNeasy Mini Kit, Qiagen, Valencia, CA, USA) according to the manufacturer’s instructions. Extracted RNA was stored at −80°C. The forward and reverse primer pairs used to amplify the approximately 1,000 bp region of H-gene were synthesized based on primer pair 7 (44). A single-step process was used for cDNA production and PCR amplification in this case using a commercially available master mix (SuperScript III Platinum One-Step RT-PCR kit, Invitrogen, Life Technologies, Grand Island, NY, USA). For RT-PCR, 2 µL of extracted RNA per sample was run in 25 µL total volume reactions using 300 nM of each primer and one unit of RNAse inhibitor (RNAse Out, Invitrogen, Life Technologies, Grand Island, NY, USA). Samples were amplified in a thermal cycler with an RT step at 50°C for 30 min, an activation step at 94°C for 2 min, followed by 35 cycles of denaturation at 94°C for 30 s, annealing at 60°C for 1 min, and elongation at 72°C for 3 min, with an additional elongation step at 72°C for 10 min. The RT-PCR products were electrophoresed on a 2% TAE agarose gel stained with SYBR Safe and visualized. Products with a single band at ~1,000 bases were purified using QIaquick PCR purification kit (Affymetrix, Santa Clara, CA, USA). All products were capillary sequenced at the Eurofins Genomics, KY, USA, using the same primers as in the PCR reactions. Chromatograms were edited and assembled using Geneious software. This involved taking raw chromatogram results for forward and reverse primer reads and trimming the ends to remove poor-quality regions. The error probability limit was set to 0.05 to trim bases with quality score less than ~13. Next, forward and reverse pairs were assembled using the *de novo* assembly feature. Finally, a consensus sequence was generated for each pair. Further available H-gene sequences from the USA were downloaded from GenBank and aligned in Geneious with the study isolates using the MUSCLE algorithm (45) (the full list of isolates from this study is listed in Table S2).

#### Phylogenetic tree estimation

A bootstrap consensus phylogenetic tree of the alignment of 88 CDV sequences (56 from Genbank reference sequences and 32 from the present study) was estimated with IQtree using the GTR + F + G4 as the best-fitted substitution model (according to Bayesian Information Criteria in ModelFinder),(46). Bootstrap values associated with internal nodes were estimated from 1,000 iterations (46, 47).

### Phylogenetic exploration of temporal signal in the 32 CDV isolates collected in our study

The sequences under investigation were checked for a sufficient “temporal signal” using the software Tempest (48). Tempest uses a “non-clock” phylogenetic tree (where branch lengths are scaled as genetic distances) (Fig.S4) and computes a linear regression between root-to-tip distance and the sampling time for each tip (Fig.S4). A strong linear relationship would suggest that molecular differences between species pairs are proportional to the time of their separation. The phylogenetic tree used for this analysis was a neighbor-joining tree generated in R using the package ape (49), rooted to minimize the residual mean squares of a linear regression of sampling times against root-to-tip distance (Fig.S4A).

## RESULTS

### Canine distemper prevalence and spatial pattern

**Fig 1:**
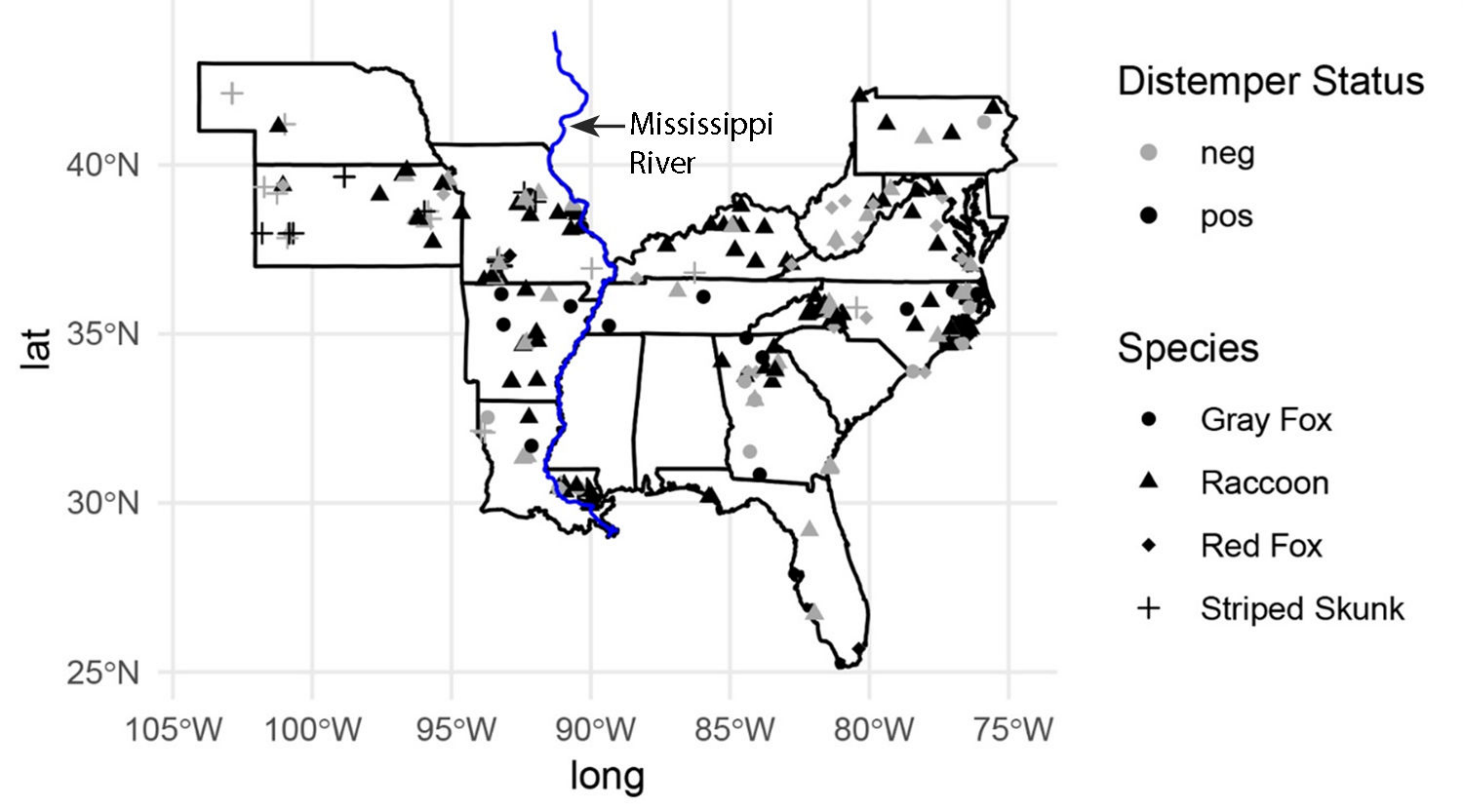
Map of states from which necropsy cases of mesocarnivore species were analyzed for canine distemper virus infection from January 2019 to December 2022 (*n* = 270). The location of Mississippi River is blue continuous line and shown by the arrow.

**Fig 2:**
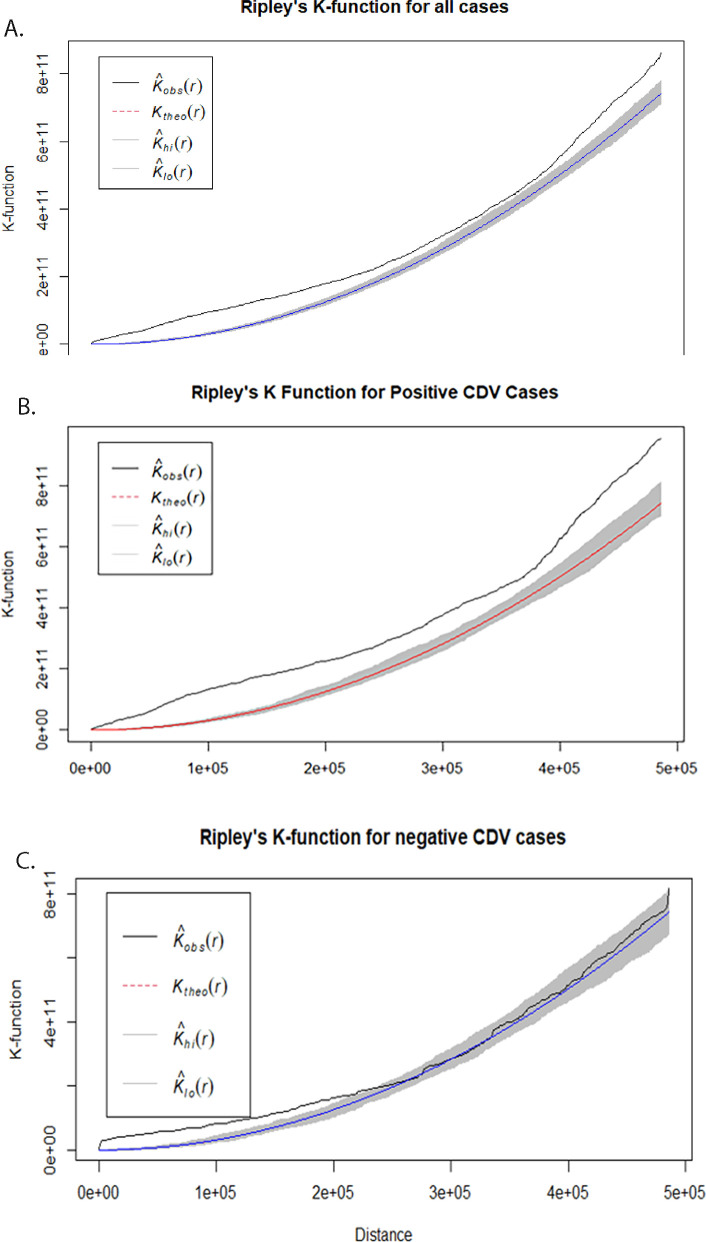
Spatial clustering analysis based on Ripley’s K-function of cases of select mesocarnivore species submitted to Southeastern Cooperative Wildlife Disease Study from January 2019 to December 2022. Plot A: Entire data set. Plot B: CDV-positive cases. Plot C: CDV-negative cases. *r* (the *x*-axis) represents distance from a point and *K*(*r*) represents the K-function. 𝐾̂ 𝑜𝑏𝑠(𝑟) (solid line) is the Ripley’s K-statistic for the observed cases. 𝐾𝑡ℎ𝑒𝑜(𝑟) (dashed line) is the K-statistic for a completely random (Poisson) point process (no spatial clustering). 𝐾̂ ℎ𝑖 (𝑟) and 𝐾̂ 𝑙𝑜(𝑟) (shading around dashed line) are the upper and lower confidence interval envelopes for the Poisson simulation*.* When the solid line of observed data points is greater than the dashed line and error bands (gray area), then there is spatial clustering of cases (case clustering is greater than a random distribution). When the solid line of observed data points is within the random K-statistic, then spatial clustering is similar to random.

A total of 270 mesocarnivores underwent postmortem evaluation at SCWDS from January 2019 to December 2022. At necropsy, 158 out of 270 mesocarnivores (58.5%) were diagnosed with CDV infection (Fig. 1) by histopathology and DFA test and/or IHC. There were four host species represented in these data that had positive CDV cases: raccoon, gray fox, striped skunk (*Mephitis mephitis*), and red fox (*Vulpes vulpes*). These animals originated from 13 different states. The state and species distributions are shown in Table 1. The entire data set for all necropsy cases, including those diagnosed with canine distemper as well as those from which CDV was not detected, showed spatial clustering with Ripley’s K analysis (Fig. 2). This pattern was most obvious at shorter distances, whereas the plot almost returned to being randomly spatially distributed at greater distances (Fig. 2, Plot A). The distemper cases (Fig. 2, Plot B) showed a greater degree of clustering, especially at larger distances compared to the entire data set (Fig. 2, Plot A). CDV-negative cases only had a small degree of clustering at short distances while at larger distances, the clustering was similar to random (Plot C).

**TABLE 1:** Summary of necropsy cases of select mesocarnivore by species and state submitted to the Southeastern Cooperative Wildlife Disease Study from January 2019 to December 2022

State	Gray fox	Raccoon	Red fox	Striped skunk	Total
Arkansas	3	14	0	0	17
Florida	0	6	2	0	8
Georgia	9	25	3	0	37
Kansas	0	14	3	12	29
Kentucky	0	18	1	1	20
Lousiana	4	14	1	3	22
Missouri	2	19	2	9	32
North Carolina	15	53	3	2	73
Nebraska	0	1	0	2	3
Pennsylvania	1	5	0	0	6
Tennessee	2	1	0	0	3
Virginia	0	3	3	0	6
West Virginia	0	9	5	0	14
Total	36	182	23	29	270

### Generalized linear model of canine distemper diagnosis

The results of the model showed surface imperviousness, precipitation, elevation, age, and species to be the most statistically significant (*P* < 0.01) explanatory variables in the model (Table 2). The full results of the model analysis for all explanatory variables are shown in Table S4. The best-fitting model from the data is described below:

Let *Y* represent the probability of distemper diagnosis in mesocarnivore necropsy case submissions (a binomial response variable)


$$logit\left(P\left(Y\;=\;1\right)\right)\;=\;\beta_{0}\;+\;\beta_{1}\cdot Species\;+\;\beta_{2}\cdot Age\;+\;\beta_{3}\cdot Month\;+\;\beta_{4}\cdot Latitude\;+\;\beta_{5}\cdot knn.dist\;+\;\beta_{6}\cdot Elevation\;+\;\beta_{7}\cdot Precipitation\;+\;\beta_{8}\cdot Temperature\;+\;\beta_{9}\cdot Imperviousness\;+\;\beta_{10}\cdot Description\;Land\;Use\;+\;\beta_{11}\cdot \left(Species\;\times \;knn.dist\right)\;+\;\beta_{12}\cdot \left(Species\;\times \;Temperature\right)\;+\;\beta_{13}\cdot \left(Latitude\;\times \;Elevation\right)\;+\;\beta_{14}\cdot \left(Elevation\;\times \;Imperviousness\right)\;+\;\beta_{15}\cdot \left(Age\;\times \;Month\right)$$

**TABLE 2:** Summary statistics for significant (*P* < 0.01) explanatory variables for the GLM, ordered by absolute value of the standardized coefficient (Std_coef)^*a*^

Explanatory variable	Estimate	2.50%	97.50%	Std. error	*z* Value	*Pr*(>|*z*|)^*b*^	Std_coef
Species raccoon: temperature	6.8487	3.9979	10.8176	1.7216	3.978	**0.0001**	3.978
Species raccoon	−117.4854	−186.61	−67.701	30.1053	−3.9025	**0.0001**	−3.9025
Elevation: imperviousness	−0.0005	−0.0008	−0.0003	0.0001	−3.8565	**0.0001**	−3.8565
lat: elevation	0.0089	0.0049	0.0143	0.0024	3.7209	**0.0002**	3.7209
Elevation	−0.2851	−0.4668	−0.1507	0.0801	−3.5586	**0.0004**	−3.5586
Precipitation	0.0098	0.0049	0.016	0.0028	3.5207	**0.0004**	3.5207
Species striped skunk	−151.1541	−247.96	−75.273	43.600	−3.4664	**0.0005**	−3.4664
Species striped skunk: temperature	9.6782	4.5707	16.3482	2.9883	3.2386	**0.0012**	3.2386
Species striped skunk: knn.dist	−0.0003	−0.0005	−0.0002	0.0001	−3.2328	**0.0012**	−3.2328
Imperviousness	0.1588	0.0564	0.2806	0.0562	2.8274	**0.0047**	2.8274

^*a*^ A full summary of the model is in Table S1. Interaction terms are those separated by a colon.

^*b*^ The Pr(>|z|) is the p-value associated with the value in the z Value column. Significance is (∝<0.01).

**Fig 3:**
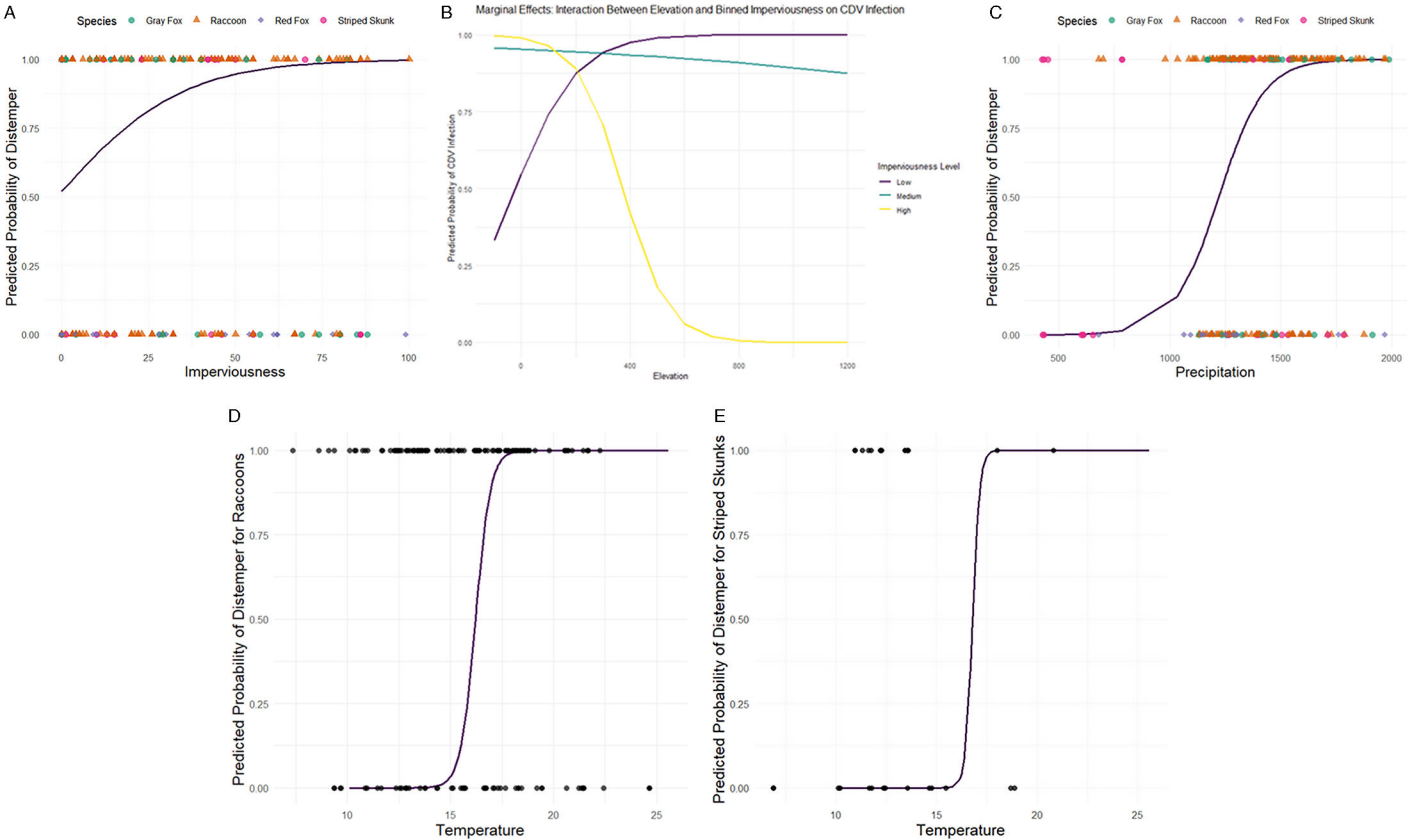
Marginal effects plots of predicted CDV infection probabilities for significant predictors in the best-fit model. (A) Predicted effects of impervious surface on CDV infection probabilities with data points from this study. (B) Predicted effects of the interaction between impervious surface and binned elevation values on CDV infection probability. (C) Predicted effects of precipitation on CDV infection probability with data points from this study. (D) Predicted effects of the interaction between raccoons, temperature, and CDV infection probability with data points from this study. (E) Predicted effects of the interaction between striped skunks and CDV infection probability with data points from this study.

The model improvements throughout the fitting process are summarized in Table 3; the full list of models is included in Table S3. Model testing showed a prediction accuracy of the best-fit model for canine distemper cases 0.61. The model had a precision of 0.56 and a recall of 0.75 with an F1 score of 0.64. The best-fit model performed adequately based on standard diagnostics, such as influence plots, residuals versus fitted values, Normal *Q*-*Q* plots of theoretical versus sample quantiles, and DHARMa diagnostic plots (Fig. S1 and S2). Forest plots of standardized residuals for all model predictors are in Fig. S3. Figure 3 shows marginal effects plots of predicted CDV infection probabilities for significant predictors in the best-fit model.

**TABLE 3:** Summary of the improvements to the model metrics throughout the GLM fitting process^*a*^

Model	Residual deviance	Residual d.f.	AIC	Delta AIC	AIC weight
Global model	179.05	173	237.05	87.46	1.02 × 10^–19^
Interactions	101.37	163	179.37	29.78	3.41 × 10^–7^
Backwards selection	103.17	168	175.17	25.58	2.79 × 10^–6^
Outliers	77.59	165	149.59	–	–
Best-fit	77.59	165	149.59	–	–

^*a*^ -, Refers to the reference (best-fit) model therefore Delta-AIC and AIC weight not shown.

### CDV phylogeny

**Fig 4:**
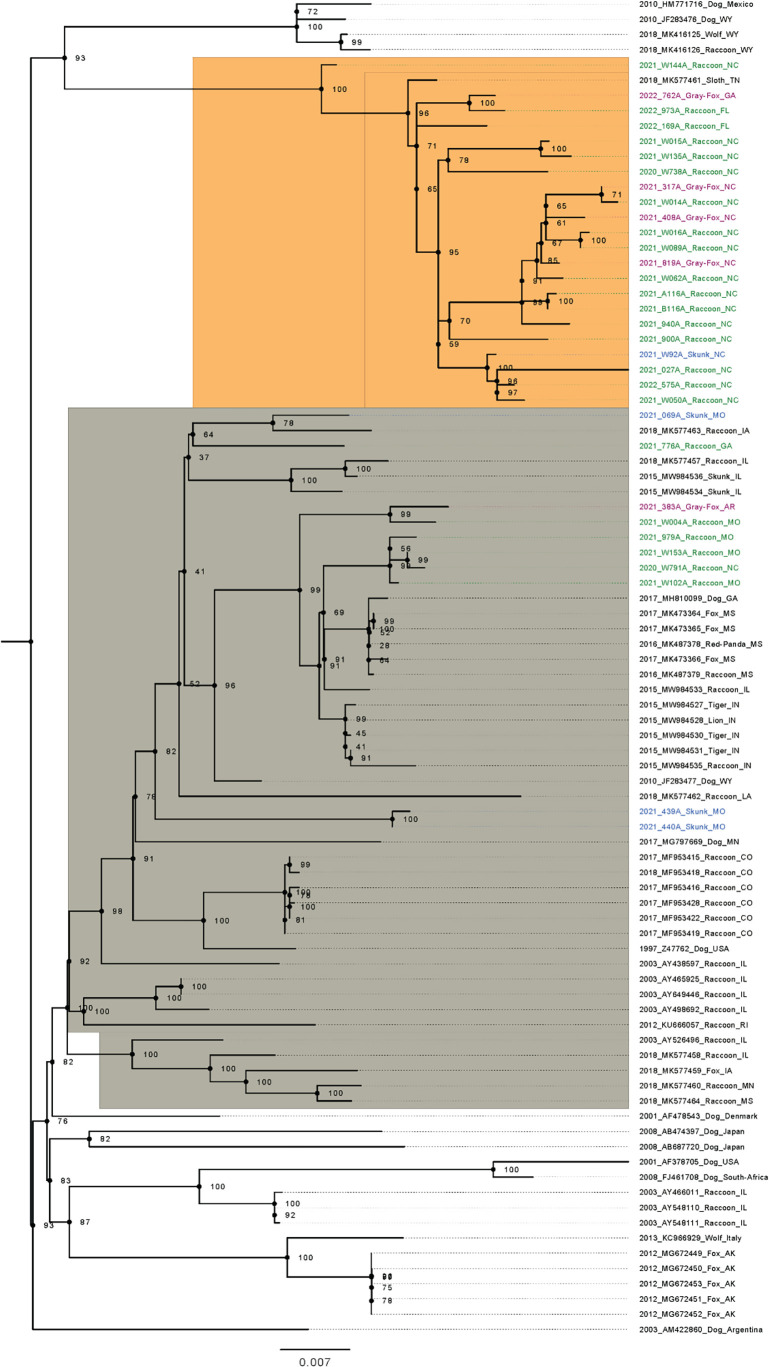
Phylogenetic tree for H-gene of canine distemper virus isolates from necropsy cases of mesocarnivore species submitted to the Southeastern Cooperative Wildlife Disease Study from January 2019 to December 2022. This study produced isolates in colored text. Fuchsia, blue, and red color isolates correspond to isolates collected from gray foxes, skunks, and raccoons, respectively. Those in black text were accessed from Genbank. The orange-highlighted clade is the proposed eastern group of viruses isolated in the study from CDV cases in states east of the Mississippi River (NC, GA, and FL). The gray shading corresponds to the western group with viruses from AR, MO, and CO.

A total of 32 CDV partial H-gene (~1,200 bp) isolates from five states from this study (Georgia [GA], Florida [FL], North Carolina [NC], Missouri [MO], and Arkansas [AR]), in addition to 56 additional sequences downloaded from GenBank, were aligned and used to build a phylogenetic tree. Table S5 provides the Genbank accession number and sequence results of the 32 isolates. This analysis was conducted to understand evolutionary relationships between the CDV isolates found in this study and other available isolates from across the US. After tree inspection, the sequences from this study fell into two distinct clades. A total of 22 the isolates from the eastern states (NC, FL, and GA) clustered together. A total of five isolates from MO and AR (i.e., west of the Mississippi River) clustered distinctly from this large eastern clade, although one (NC 2020 791A Raccoon NC) and one GA isolate (2021_776A Raccoon GA) grouped with these more western isolates (Fig. 4). This western clade had moderate bootstrap support of 64.6.

The plot of the root-to-tip genetic distance against sampling time showed that the sampling dates did not effectively explain the diversity found in the partial H-gene sequences (R^2^ = 0.033), which suggests that the data set has no temporal structure (Fig. S4).

## DISCUSSION

This study shows a relatively high overall diagnosis of CDV infections in submitted mesocarnivores. Additionally, a generalized linear model for wild mesocarnivores diagnosed with canine distemper showed that surface imperviousness, precipitation, belonging to the adult age class, and species were significant positive explanatory variables, while elevation had a significant negative association with the likelihood of the animal having canine distemper. Ripley’s K analysis showed more spatial clustering of canine distemper cases than negative cases, particularly at larger distances. This follows the ecology of the disease as it is a directly transmitted pathogen. Hence, an outbreak spreads and is maintained through an in-contact, susceptible population, resulting in case clustering. There is some spatial autocorrelation in the data at shorter distances, which is to be expected given the passive nature of sample collection. In addition, citizens reporting and/or biologists submitting cases have a radius within which they operate with a gap distance until the next person, resulting in this clustering pattern.

Surface imperviousness is a significant explanatory variable in the model, with a positive relationship between increased imperviousness and the likelihood of being diagnosed with canine distemper, suggesting that land use plays a role in the disease ecology of canine distemper. Higher surface imperviousness generally corresponds to areas of greater human development (50). There are several reasons why these more impervious areas, typical of urban or urbanizing landscapes, result in a higher likelihood of CDV infection in wild mesocarnivores. Urban areas often possess abundant resources for anthropophilic species, such as the raccoon (51). These resources may be less prone to seasonal fluctuations and include supplemental food sources (e.g., household waste, supplemental outdoor feeding of cats or wildlife) and shelter. As a result, urban and suburban areas can support much greater raccoon population densities (12) than more natural habitats. In addition to more abundant resources in urban areas, there tends to be greater aggregation of resources. This clumping of resources, for example, at a large landfill site or deliberate food placed for feral cats or other wildlife, results in two factors that are of importance in disease transmission: migration of individuals into the area and exceptionally high intra- and interspecific contact rates (52). Contact rates play a vitally important role in distemper disease spread, with higher population density resulting in greater contact rates and, consequently, higher rates of pathogen transmission (53). One study showed that higher population density in response to resource availability resulted in higher parasite richness and increased prevalence of the zoonotic nematode *Baylisascaris procyonis* in raccoons (54). In urban dwelling feral cats, there was a higher prevalence of severe fever with thrombocytopenia syndrome virus antibodies in urban dwelling feral cats than in their rural counterparts (55). Canine distemper cases are more prevalent in urban and suburban counties than rural counties in parts of the US that support a much lower population density of raccoons (27).

Urbanization can also influence interspecific and intraspecific behavioral interactions, affecting disease transmission. For instance, bobcats and pumas may exhibit different behaviors in urban versus rural settings, which can increase contact and interspecific interactions, potentially facilitating cross-species infectious diseases like lentivirus (56, 57). Conversely, some highly urbanized areas may reduce pathogen spread; phylogenetic analyses indicate that directly transmitted pathogens, such as feline immunodeficiency virus in bobcats, may spread more quickly in natural habitats (58). Studies that integrate host and pathogen genetics to explore mesocarnivore behavior across urban-rural gradients could enhance our understanding of CDV maintenance in wildlife.

Furthermore, there is the question of how the quality of more urbanized diets (59) affects these individuals' metabolism and immune response and whether this may also result in increased pathogen shedding and disease susceptibility. Theoretical studies also have shown that resource provisioning can have significant effects on pathogen prevalence in urban environments (60). Prevalence of directly transmitted parasites was shown to respond more strongly to provisioning, which has direct consequences on our study system, as CDV is a directly transmitted pathogen (61). There is also the possibility that closer contact with domestic dogs in urbanized areas results in more CDV spillover events into wildlife populations than in rural areas, as CDV spillover from dogs into wildlife is often reported (28, 62).

The model also revealed a significant interaction between imperviousness and elevation, with a negative relationship between these factors and CDV infection. This is likely because impervious surfaces at high elevations correspond to stony surfaces in more natural vs. developed areas. This implies that as a single variable, elevation is negatively associated with CDV infection (i.e., wild mesocarnivores at higher elevations are less likely to contract CDV infection). As previously discussed, this is a density-dependent disease with higher population densities producing larger outbreaks and being more able to sustain outbreaks. At higher elevations, the population densities of mesocarnivores tend to be significantly lower for several reasons, such as less suitable habitat, harsher environmental conditions (e.g., extreme temperatures), and less food availability (63). However, at lower elevations, there is a positive association between impervious surfaces and CDV infections, possibly due to increased mesocarnivore densities and increased intraspecific and interspecific contact.

Increased precipitation also had a significant positive association with CDV infection. This can be explained from both the standpoint of the virus and the host. Higher rainfall may increase humidity within the study area, which in turn may facilitate aerosol transmission, as occurs with CDV (64). Furthermore, precipitation might soften and disperse fresh urine or feces, which may contain infectious particles. It is possible that susceptible animals will step into fresh feces or infectious urine, get it on their footpads, and then clean their footpads, potentially ingesting the virus. Additionally, increased humidity may prolong virus survival on fomites, allowing transmission to new susceptible hosts (65). From the hosts’ behavior standpoint, increased precipitation may lead to increased denning behaviors, resulting in increased intraspecific contact and, thus, CDV transmission. Furthermore, increased precipitation may favor key reservoir host occupancy in species such as raccoon (66) and could increase transmission due to increased resources in areas of higher precipitation, leading to increased intraspecific and interspecific contact.

There was a significant positive association between predicted CDV infection rates and mean annual temperature/species interaction for raccoons and striped skunks. This observation may be due to increased raccoon habitat suitability in areas of higher mean annual temperatures (66). At higher mean annual temperatures, raccoons may be more active, and come into more intraspecific and interspecific contact, thus driving increased CDV infection. Locations with higher mean annual temperatures may result in higher resource availability for raccoons and striped skunks, potentially driving up relative abundance, density, and, subsequently, CDV transmission.

While the species of mesocarnivore was significantly associated with distemper diagnosis in our model, the size of the error in these cases warrants caution in this interpretation and a larger data set is needed to evaluate this hypothesis. Because our data were subdivided by species, the sample sizes were small for foxes and skunks (*N* < 40). Regardless, among species, gray foxes had the highest predicted CDV infection rates, raccoons and striped skunks had a significantly lower overall predicted CDV infection probability (raccoons slightly more likely to be infected than striped skunks), with red foxes having the lowest predicted infection rates across our study area. There may be either ecological or immunological reasons for these observations. Data from Pennsylvania in the Northeastern US (67) show significantly higher CDV seroprevalence in red foxes than gray foxes, suggesting that red foxes may be more exposed to CDV (or exposed to lower viral doses of CDV through fomites) than gray foxes, but less likely to die. Emerging data suggest that gray foxes are more likely to die from the pathogenic effects of CDV infection (68). Although raccoons dominate CDV cases overall, model results suggest that gray foxes could be the most susceptible to CDV infection in our study. Raccoon density estimates range from 2.24 to 5.44 animals/km^2^ in the southeastern US, depending on habitat type (69), with striped skunks having more variable densities but typical ranges of 1.8–4.8 km^2^ (70). The similarity in skunk and raccoon densities and adaptability to a wide range of environments, similarities in reproductive and nutritional ecology (polygamous omnivores), and geographic overlap, may account for their similarities in predicted CDV infection status. Furthermore, red foxes may be less susceptible to CDV due to highly variable home range sizes, foraging, and reproductive behaviors (71), which may lessen spatial overlap and direct contact with infected raccoons or skunks, or other infected wildlife in areas where they overlap. Gray foxes, however, have smaller and less variable home ranges with a lot of shrub and tree cover and areas of human use near roads (72), which might result in them having a higher conspecific and heterospecific spatial and temporal exposure or contact in areas where outbreaks are taking place. If they truly are more susceptible to CDV, gray foxes could serve as “spillback” hosts to raccoons, connecting epizootic spread across landscapes. Further sampling of sequences for phylodynamic analysis to evaluate temporal-spatial transmission directionalities is a promising avenue for future research.

Model results show a significantly higher probability of CDV infection in adults compared to subadults and juveniles and a higher yet non-significant predicted probability of subadult infection. This observation is likely related to overlapping home ranges of males and females to allow for males to increase the probability of mating with multiple females (69), and potentially related to intraspecific aggression. Juveniles may have lower predicted infection probabilities due to (73) maternal antibodies lessening disease susceptibility or increasing the likelihood that this young age group would be killed by other means (e.g., vehicular trauma or other infectious process), which may skew the data.

Overall, there is a slight positive association between infection and the month of the year of carcass reporting, which may indicate a general trend for all age classes and species across the year, accounting for inter-specific contact as well. However, predicted negative associations of CDV infection rates with adult mesocarnivores and month overall may also be due to increased intraspecific contact earlier in the year, as the breeding season for raccoons is February to June, peaking in March, and a decrease in adult contacts later in the year (69, 74). Breeding season for striped skunks also occurs earlier in the year (February to April) (70).

The results of our study have implications for surveillance and conservation efforts, emphasizing that increased surveillance in urbanized areas may be important to improve our understanding of CDV transmission across urban landscapes and for earlier identification of outbreaks. From the standpoint of developing a future surveillance system, while the more specific land cover data did improve the model, the individual land cover types were not significant explanatory factors. For a streamlined risk evaluation system, the imperviousness data may be helpful. It may even be possible in these urban areas to instigate a citizen science program using a reporting application like that used for rabies in skunks in Colorado (75). This may allow for vaccination programs in the face of outbreaks threatening more vulnerable mammal species.

This study demonstrated significant genetic diversity in CDV H-gene sequences among wild mesocarnivores in our samples, which were broadly separated into groups east and west of the Mississippi River, an observation that is supported by past research from CDV isolates from wild carnivores in North America (76, 77). In our study, the phylogenetic analysis of CDV isolates west of the Mississippi River showed a distinct cluster from those of wild mesocarnivores in states east of the Mississippi River. Most NC, GA, and FL isolates clustered closely within the phylogenetic tree. Isolates from more western states, AR and MO, clustered separately from these eastern isolates and in a less clearly defined group. This suggests that all the eastern isolates are closely related. Given that the states of GA, FL, and NC are in close geographic proximity, these isolates may have originated from a large regional outbreak.

Second, the Mississippi River forms a much more difficult barrier for the virus to traverse than smaller rivers. This is consistent with typical CDV transmission mechanisms, as vectors and birds do not transmit the virus, and the virus does not undergo airborne transmission, so it would need to be brought across the river by an infected mammal. This may have resulted in distinct strains evolving on each side of the river. The Mississippi River, as a barrier to disease dispersal in wildlife, has been demonstrated for rabies (78). However, based on our study and others, it is not an impenetrable barrier, as there was one GA isolate and one NC isolate that clustered with isolates from west of the Mississippi River.

Several ecological and/or anthropogenic factors may explain why we identified eastern variants to the west of the Mississippi River. Coyotes, although unsampled in this study, could be a source of the western presence of variants found primarily east of the Mississippi River because long-distance movements of coyotes in the Southeastern US have been documented up to 393 km west of the location where they were radio-collared (79). Furthermore, the Mississippi River is not an impermeable geographic barrier, as mesocarnivores can use bridges, swim, and float on flotsam across river barriers. Domestic dogs that transmit CDV might spread the virus across state lines and the Mississippi River by long-distance movement or human transport. Additionally, long-distance transport of wild CDV reservoirs by hunters or wildlife rehabilitators could introduce CDV strains to new areas. Long-distance translocation of a raccoon from Southeastern New York State led to a rabies outbreak in Ontario (80). Additionally, there could be unintentional long-distance travel of raccoons hiding on commercial transport (e.g., trucks and cargo trains) (81). Therefore, CDV strain translocation could be an unintended consequence of unintentional or intentional long-distance mesocarnivore movement or translocation (82).

### Limitations

This study’s limitations apply to sampling biases and the fact that animals in our study were not randomly sampled. The samples were collected passively through opportunistic submissions to SCWDS by state wildlife agencies and federal government partners (e.g., U.S. Fish and Wildlife Service), across the southeastern US. Thus, sample acquisition depends on numerous factors: a dead or ill animal being reported to the authorities (often by concerned members of the public), a dead or ill animal being seen by the authorities, and a willingness by the authorities to submit the animal for necropsy. This undoubtedly excludes many affected animals that are either not reported, not seen (e.g., in more remote or natural habitats), or not submitted for testing. Additionally, areas with rabies concerns are likely to submit more cases as the two diseases present similarly from a clinical standpoint. As such, cases commonly are assessed for both viruses (i.e., CDV and rabies virus), with occasional documentation of coinfection (83). In cases with human exposure to a neurologic mesocarnivore, which require immediate submission to the respective state’s rabies laboratory for diagnostic testing, it is possible that some CDV-positive animals missed detection as they were not submitted to SCWDS. Another limitation of our study is the fact that we were unable to do RT-PCR and sequencing on all CDV-diagnosed cases due to the lack of available funding. The raster (i.e., land cover) data from NLCD are from 2019, which is before most of our samples were collected; however, land cover is unlikely to have changed much in this period as the effects of this time lag are likely minimal, especially due to the economic impact of the COVID-19 pandemic. Although the GLM and phylogenetic tree may indicate some potential relationships between land use and CDV in the case of the GLM, and geographically in the case of the tree, neither model had very strong statistical support, both in the 0.60–0.70 range. Testing of these hypotheses with a larger and more comprehensive data set combined with targeted surveillance would be an important next step in attempting to reveal more about these potential relationships and developing models with stronger support.

### Conclusion

The conclusions of this study are twofold. First, it provides further evidence of widespread CDV infection in wild mesocarnivores within the southeastern US and shows that there is significant CDV genetic diversity in the region, particularly divided by the Mississippi River. Second, results suggest that, with precipitation and temperature, human land use may play an important role in the disease ecology of this virus, with wild carnivores in areas of intense human development at higher risk for CDV infection. Land use change is a complex challenge regarding disease dynamics at the wildlife-domestic-human interface, with no solution that fits every disease. Social responsibility and responsible urban planning integrated with biodiverse and ecologically resilient landscapes can mitigate future problems. Additionally, surveillance and control measures, such as vaccination (84), particularly regarding diseases in synanthropic species also can play a crucial role in the dynamics of wildlife disease in urban environments.

## Data Availability

Metadata and code used for the evaluation of canine distemper spatial patterns and statistical analyses, data visualization, and the list of ID numbers and Genbank accession numbers used in the phylogenetic analyses are available at Figshare at the following link: https://doi.org/10.6084/m9.figshare.27308337. Table S5 shows Genbank accession numbers, ID numbers, and sequence data for the partial H-gene sequence done in this study. Sequences for all the data used in the phylogenetic analysis are identified by accession number and available at https://www.ncbi.nlm.nih.gov/genbank/.

## SUPPLEMENTAL MATERIAL

Supplemental material spectrum.01225-24-s0001.docx Tables S1 to S5; Fig. S1 to S4.

ASM does not own the copyrights to Supplemental Material that may be linked to, or accessed through, an article. The authors have granted ASM a non-exclusive, world-wide license to publish the Supplemental Material files. Please contact the corresponding author directly for reuse.
