# Supplementary material for: Canine distemper virus phylogenetic structure and ecological correlates of infection in mesocarnivores across anthropogenic land use gradients
Source: Microbiol Spectr. 2025 Mar 3;13(4):e01225-24. doi: 10.1128/spectrum.01225-24 (PMC11960092; doi:10.1128/spectrum.01225-24)
Supplement: Supplemental material — Tables S1 to S5; Fig. S1 to S4. [file spectrum.01225-24-s0001.docx]

Supplementary Materials

**Table S1: National Land Cover Database Class Legend and Description.** *Detailed descriptions of land cover classes designated to each pixel of the national land cover* *database for the United States. Original source: https://*[*www.mrlc.gov/data/legends/national-*](http://www.mrlc.gov/data/legends/national-) *land-cover-database-class-legend-and-description.*

**National Land Cover Database Class Legend and Description**

| **Class\ Value**  **Water** | **Classification Description** |
| --- | --- |
| 11 | **Open Water**- areas of open water, generally with less than 25% cover of vegetation or soil. |
| 12 | **Perennial Ice/Snow**- areas characterized by a perennial cover of ice and/or snow, generally greater than 25% of total cover. |
| **Developed** |  |
| 21 | **Developed, Open Space**- areas with a mixture of some constructed materials, but mostly vegetation in the form of lawn grasses. Impervious surfaces account for less than 20% of total cover. These areas most commonly include large-lot single-family housing units, parks, golf courses, and vegetation planted in developed settings for recreation, erosion control, or aesthetic purposes. |
| 22 | **Developed, Low Intensity**- areas with a mixture of constructed materials and vegetation. Impervious surfaces account for 20% to 49% percent of total cover. These areas most commonly include single-family housing units. |
| 23 | **Developed, Medium Intensity** -areas with a mixture of constructed materials and vegetation. Impervious surfaces account for 50% to 79% of the total cover. These areas most commonly include single-family housing units. |
| 24 | **Developed High Intensity**-highly developed areas where people reside or work in high numbers. Examples include apartment complexes, row houses and commercial/industrial. Impervious surfaces account for 80% to 100% of the total cover. |
| **Barren** | |
| 31 | **Barren Land (Rock/Sand/Clay)** - areas of bedrock, desert pavement, scarps, talus, slides, volcanic material, glacial debris, sand dunes, strip mines, gravel pits and other accumulations of earthen material. Generally, vegetation accounts for less than 15% of total cover. |
| **Forest** | |
| 41 | **Deciduous Forest**- areas dominated by trees generally greater than 5 meters tall, and greater than 20% of total vegetation cover. More than 75% of the tree species shed foliage simultaneously in response to seasonal change. |
| 42 | **Evergreen Forest**- areas dominated by trees generally greater than 5 meters tall, and greater than 20% of total vegetation cover. More than 75% of the tree species maintain their leaves all year. Canopy is never without green foliage. |

| 43 **Mixed Forest**- areas dominated by trees generally greater than 5 meters tall, and greater than 20% of total vegetation cover. Neither deciduous nor evergreen species are greater than 75% of total tree cover. | |
| --- | --- |
| **Shrubland** | |
| 51 | **Dwarf Scrub**- Alaska only areas dominated by shrubs less than 20 centimeters tall with shrub canopy typically greater than 20% of total vegetation. This type is often co-associated with grasses, sedges, herbs, and non-vascular vegetation. |
| 52 | **Shrub/Scrub**- areas dominated by shrubs; less than 5 meters tall with shrub canopy typically greater than 20% of total vegetation. This class includes true shrubs, young trees in an early successional stage or trees stunted from environmental conditions. |
| **Herbaceous** | |
| 71 | **Grassland/Herbaceous**- areas dominated by gramanoid or herbaceous vegetation, generally greater than 80% of total vegetation. These areas are not subject to intensive management such as tilling, but can be utilized for grazing. |
| 72 | **Sedge/Herbaceous**- Alaska only areas dominated by sedges and forbs, generally greater than 80% of total vegetation. This type can occur with significant other grasses or other grass like plants, and includes sedge tundra, and sedge tussock tundra. |
| 73 | **Lichens**- Alaska only areas dominated by fruticose or foliose lichens generally greater than 80% of total vegetation. |
| 74 | **Moss**- Alaska only areas dominated by mosses, generally greater than 80% of total vegetation. |
| **Planted/Cultivated** | |
| 81 | **Pasture/Hay**-areas of grasses, legumes, or grass-legume mixtures planted for livestock grazing or the production of seed or hay crops, typically on a perennial cycle. Pasture/hay vegetation accounts for greater than 20% of total vegetation. |
| 82 | **Cultivated Crops** -areas used for the production of annual crops, such as corn, soybeans, vegetables, tobacco, and cotton, and also perennial woody crops such as orchards and vineyards. Crop vegetation accounts for greater than 20% of total vegetation. This class also includes all land being actively tilled. |
| **Wetlands** | |
| 90 | **Woody Wetlands**- areas where forest or shrubland vegetation accounts for greater than 20% of vegetative cover and the soil or substrate is periodically saturated with or covered with water. |
| 95 | **Emergent Herbaceous Wetlands**- Areas where perennial herbaceous vegetation accounts for greater than 80% of vegetative cover and the soil or substrate is periodically saturated with or covered with water. |

**Table S2. List of CDV partial H-gene isolates generated in wild mesocarnivores diagnosed with canine distemper at the Southeastern Cooperative Wildlife Disease Study from January 2019 to December 2022 and their sequence quality metrics.**

| Isolate Name | % GC | % HQ | %  Identical Sites | % LQ | % MQ | %  Pairwise Identity | At least Q20 | At least Q30 | At least Q40 | Mean Coverage | Sequence Length |
| --- | --- | --- | --- | --- | --- | --- | --- | --- | --- | --- | --- |
| 027A_Raccoon_NC | 44.00 | 79.20 | 99.80 | 9.3 | 11.5 | 99.80 | 90.70 | 83.44 | 79.19 | 1.707965 | 1129 |
| 069A_Skunk_MO | 41.90 | 92.70 | 95.30 | 2.0 | 5.40 | 95.40 | 98.04 | 96.16 | 92.69 | 1.268032 | 1121 |
| 169A_Raccoon_FL | 43.90 | 95.00 | 99.10 | 1.7 | 3.30 | 99.10 | 98.31 | 96.17 | 95.01 | 1.902852 | 1122 |
| 317A_Gray_Fox_NC | 43.70 | 92.80 | 99.60 | 3.2 | 4.00 | 99.60 | 96.81 | 94.85 | 92.81 | 1.631766 | 1127 |
| 383A_Gray_Fox_AR | 42.50 | 95.10 | 99.10 | 1.6 | 3.30 | 99.10 | 98.40 | 96.62 | 95.11 | 1.911922 | 1124 |
| 408A_Gray_Fox_NC | 43.90 | 94.20 | 99.80 | 1.7 | 4.10 | 99.80 | 98.32 | 96.45 | 94.24 | 1.757092 | 1128 |
| 439A_Skunk_MO | 42.90 | 90.10 | 95.20 | 1.3 | 8.60 | 95.30 | 98.74 | 94.97 | 90.13 | 1.298923 | 1114 |

| **Isolate Name** | **% GC** | **% HQ** | **%**  **Identical Sites** | **% LQ** | **% MQ** | **%**  **Pairwise Identity** | **At least Q20** | **At least Q30** | **At least Q40** | **Mean Coverage** | **Sequence Length** |
| --- | --- | --- | --- | --- | --- | --- | --- | --- | --- | --- | --- |
| 440A_Skunk_MO | 42.70 | 93.90 | 99.60 | 1.2 | 4.90 | 99.60 | 98.84 | 96.17 | 93.94 | 1.654189 | 1122 |
| 575A_Raccoon_NC | 43.40 | 96.20 | 99.20 | 1.0 | 2.80 | 99.20 | 99.02 | 97.32 | 96.25 | 1.780161 | 1119 |
| 762A_Gray_Fox_GA | 44.00 | 95.70 | 99.50 | 1.4 | 2.80 | 99.50 | 98.58 | 97.33 | 95.73 | 1.9065 | 1123 |
| 776A_Raccoon_GA  = | 42.10 | 94.60 | 96.10 | 1.5 | 3.90 | 96.10 | 98.49 | 96.98 | 94.58 | 1.452529 | 1125 |
| 819A_Gray_Fox_NC | 44.50 | 89.90 | 92.80 | 1.6 | 8.50 | 93.30 | 98.35 | 94.42 | 89.85 | 1.178245 | 1094 |
| 900A_Raccoon_NC | 43.50 | 92.60 | 96.60 | 3.2 | 4.20 | 96.70 | 96.79 | 94.21 | 92.61 | 1.577265 | 1123 |
| 940A_Raccoon_NC | 43.30 | 92.50 | 98.60 | 3.6 | 3.90 | 98.70 | 96.38 | 94.44 | 92.50 | 1.383936 | 1133 |
| 973A_Raccoon_FL | 43.50 | 96.40 | 98.90 | 1.3 | 2.20 | 98.90 | 98.66 | 97.41 | 96.42 | 1.740608 | 1118 |
| **Isolate Name** | **% GC** | **% HQ** | **%**  **Identical Sites** | **% LQ** | **% MQ** | **%**  **Pairwise Identity** | **At least Q20** | **At least Q30** | **At least Q40** | **Mean Coverage** | **Sequence Length** |
| 979A_Raccoon_MO | 41.90 | 95.50 | 99.00 | 1.6 | 2.90 | 99.00 | 98.39 | 96.60 | 95.53 | 1.438283 | 1118 |
| A116A_Raccoon_NC | 43.60 | 94.00 | 95.60 | 1.9 | 4.10 | 95.70 | 98.13 | 95.91 | 94.05 | 1.618037 | 1126 |
| B116A_Raccoon_NC | 43.80 | 95.20 | 97.70 | 1.5 | 3.30 | 97.70 | 98.49 | 96.81 | 95.21 | 1.579646 | 1128 |
| W004A_Raccoon_MO | 42.40 | 96.00 | 98.10 | 1.5 | 2.50 | 98.10 | 98.49 | 97.24 | 96.00 | 1.963523 | 1124 |
| W014A_Raccoon_NC | 43.90 | 92.80 | 98.10 | 2.7 | 4.40 | 98.10 | 97.26 | 94.87 | 92.83 | 1.552608 | 1130 |
| W015A_Raccoon_NC | 44.00 | 94.60 | 97.10 | 1.5 | 3.90 | 97.20 | 98.48 | 96.70 | 94.55 | 1.528571 | 1120 |
| W016A_Raccoon_NC | 43.50 | 96.60 | 99.50 | 1.3 | 2.00 | 99.60 | 98.67 | 97.42 | 96.62 | 1.949288 | 1124 |
| W050A_Raccoon_NC | 43.80 | 82.70 | 99.90 | 9.8 | 7.50 | 99.90 | 90.18 | 86.31 | 82.70 | 1.772072 | 1110 |
| W062A_Raccoon_NC | 43.50 | 95.80 | 98.80 | 1.0 | 3.20 | 98.80 | 99.02 | 97.42 | 95.82 | 1.811388 | 1124 |
| **Isolate Name** | **% GC** | **% HQ** | **%**  **Identical Sites** | **% LQ** | **% MQ** | **%**  **Pairwise Identity** | **At least Q20** | **At least Q30** | **At least Q40** | **Mean Coverage** | **Sequence Length** |
| W089A_Raccoon_NC | 43.60 | 97.40 | 98.10 | 1.5 | 1.10 | 98.10 | 98.49 | 98.04 | 97.42 | 1.943111 | 1125 |
| W092A_Skunk_NC | 43.60 | 94.80 | 100.0 | 1.4 | 3.80 | 100.0 | 98.57 | 97.23 | 94.82 | 1.719392 | 1119 |
| W092A_Skunk_NC | 43.60 | 94.80 | 100.0 | 1.4 | 3.80 | 100.0 | 98.57 | 97.23 | 94.82 | 1.719392 | 1119 |
| W102A_Raccoon_MO | 42.00 | 95.90 | 97.10 | 1.2 | 2.90 | 97.20 | 98.84 | 97.24 | 95.91 | 1.696 | 1125 |
| W135A_Raccoon_NC | 44.10 | 93.50 | 99.50 | 3.0 | 3.50 | 99.50 | 96.97 | 95.10 | 93.49 | 1.842246 | 1122 |
| W144A_Raccoon_NC | 43.40 | 85.30 | 98.10 | 5.1 | 9.50 | 98.10 | 94.87 | 90.29 | 85.34 | 1.802336 | 1112 |
| W153A_Raccoon_MO | 42.20 | 95.60 | 96.20 | 1.2 | 3.10 | 96.20 | 98.75 | 97.06 | 95.64 | 1.820285 | 1123 |
| W738A_Raccoon_NC | 44.00 | 93.20 | 98.80 | 2.2 | 4.60 | 98.80 | 97.80 | 94.63 | 93.22 | 1.45022 | 1135 |
| W791A_Raccoon_NC | 42.20 | 92.20 | 99.80 | 3.1 | 4.60 | 99.80 | 96.88 | 93.94 | 92.25 | 1.786477 | 1122 |

**Table S3: Full list of models and summary statistics included in GLM fitting process.**

|  | Residual  Deviance | Residual  d.f. | AIC | deltaAIC | AIC weight |
| --- | --- | --- | --- | --- | --- |
| Global Model | 179.05 | 173 | 237.05 | 87.46 | 1.02x10^-19^ |
| Interactions |  |  |  |  |  |
| +Species:knn.dist | 155.84 | 170 | 219.84 | 70.25 | 5.56x10^-16^ |
| +Species:Temperature | 136.42 | 167 | 206.42 | 56.83 | 4.57x10^-13^ |
| +Latitude:Elevation | 123.28 | 166 | 195.28 | 45.69 | 1.20x10^-10^ |
| +Elevation:Imperviousness | 110.59 | 165 | 184.6 | 35.01 | 2.50x10^-8^ |
| +Age:Month | 101.37 | 163 | 179.26 | 29.78 | 3.41x10^-7^ |
| Backwards selection |  |  |  |  |  |
| -Longitude | 101.48 | 164 | 177.48 | 27.89 | 8.79x10^-7^ |
| -Sex | 101.95 | 167 | 175.95 | 26.36 | 1.89x10^-6^ |
| -Distance to water | 103.17 | 168 | 175.17 | 25.58 | 2.79x10^-6^ |
| Remove outlying data (x3) | 77.59 | 165 | 149.59 | - | - |
| Best Fit Model | 77.59 | 165 | 149.59 | - | - |

**Table S4: Full list of explanatory variables from best fit model with summary statistics ranked in order of p-value from most to least significant.**

*Note: The glm function in R uses a technique called "dummy coding" to convert categorical variables into a set of binary variables, also known as "indicator variables" or "dummy variables". This is done so that the categorical variable can be included in the model as a predictor. When a categorical variable is used in a model, it is split into one binary variable for each level of the categorical variable, with a value of 1 indicating membership in that level, and a value of 0 indicating non-membership. The summary function then displays each of these binary variables as a separate factor in the output. This allows the user to see the effect of each level of the categorical variable on the response variable.*

| **Explanatory variable** | **Estimate** | **2.50%** | **97.50%** | **Std. Error** | **z value** | **Pr(>\|z\|)** | **Std_coef** |
| --- | --- | --- | --- | --- | --- | --- | --- |
| Species Raccoon:Temperature | 6.8487 | 3.9979 | 10.8176 | 1.7216 | 3.978 | **0.0001** | 3.978 |
| Species Raccoon | -117.4854 | -186.61 | -67.701 | 30.1053 | -3.9025 | **0.0001** | -3.9025 |
| Elevation:Imperviousness | -0.0005 | -0.0008 | -0.0003 | 0.0001 | -3.8565 | **0.0001** | -3.8565 |
| lat:Elevation | 0.0089 | 0.0049 | 0.0143 | 0.0024 | 3.7209 | **0.0002** | 3.7209 |
| Elevation | -0.2851 | -0.4668 | -0.1507 | 0.0801 | -3.5586 | **0.0004** | -3.5586 |
| Precipitation | 0.0098 | 0.0049 | 0.016 | 0.0028 | 3.5207 | **0.0004** | 3.5207 |
| Species Striped Skunk | -151.1541 | -247.96 | -75.273 | 43.6056 | -3.4664 | **0.0005** | -3.4664 |
| Species Striped Skunk:Temperature | 9.6782 | 4.5707 | 16.3482 | 2.9883 | 3.2386 | **0.0012** | 3.2386 |
| Species Striped Skunk:knn.dist | -0.0003 | -0.0005 | -0.0002 | 0.0001 | -3.2328 | **0.0012** | -3.2328 |
| Imperviousness | 0.1588 | 0.0564 | 0.2806 | 0.0562 | 2.8274 | **0.0047** | 2.8274 |
| **Explanatory variable** | **Estimate** | **2.50%** | **97.50%** | **Std. Error** | **z value** | **Pr(>\|z\|)** | **Std_coef** |
| Temperature | -4.0778 | -7.8629 | -1.2702 | 1.6379 | -2.4896 | **0.0128** | -2.4896 |
| description Developed, Medium Intensity | -8.6037 | -16.019 | -2.2246 | 3.4601 | -2.4865 | **0.0129** | -2.4865 |
| description Developed, Low Intensity | -5.8969 | -10.991 | -1.4086 | 2.4027 | -2.4543 | **0.0141** | -2.4543 |
| Age Adult | 7.7666 | 0.6163 | 16.7143 | 4.2588 | 1.8237 | 0.0682 | 1.8237 |
| Age Adult: month | -0.8873 | -1.9748 | -0.018 | 0.5105 | -1.738 | 0.0822 | -1.738 |
| description Mixed Forest | 22.6758 | 7.6845 | 70.7309 | 14.5587 | 1.5575 | 0.1193 | 1.5575 |
| month | 0.6874 | -0.139 | 1.6979 | 0.4859 | 1.4146 | 0.1572 | 1.4146 |
| latitude | 0.9611 | -0.3157 | 2.4089 | 0.6795 | 1.4144 | 0.1572 | 1.4144 |
| Species Raccoon:knn.dist | 0 | -0.0001 | 0 | 0 | -1.1827 | 0.2369 | -1.1827 |
| Evergreen Forest | -6.5687 | -17.001 | 0.5007 | 5.8551 | -1.1219 | 0.2619 | -1.1219 |
| description Developed, Open Space | -0.9612 | -4.3489 | 2.4689 | 1.6872 | -0.5697 | 0.5689 | -0.5697 |
| Species Red Fox | -180.3872 | -187.09 | -173.91 | 351.0974 | -0.5138 | 0.6074 | -0.5138 |
| description Developed, High Intensity | -2.5383 | -12.759 | 7.0392 | 4.9593 | -0.5118 | 0.6088 | -0.5118 |
| Red Fox: Temperature | 8.9588 | 8.5721 | 9.3426 | 19.1672 | 0.4674 | 0.6402 | 0.4674 |
| **Explanatory variable** | **Estimate** | **2.50%** | **97.50%** | **Std. Error** | **z value** | **Pr(>\|z\|)** | **Std_coef** |
| (Intercept) | 16.5173 | -72.783 | 112.543 | 46.169 | 0.3578 | 0.7205 | 0.3578 |
| description Deciduous Forest | -0.6477 | -4.3285 | 3.2041 | 1.865 | -0.3473 | 0.7284 | -0.3473 |
| description Pasture/Hay | 21.6426 | 19.0432 | 24.1959 | 117.9761 | 0.1834 | 0.8544 | 0.1834 |
| Age Subadult:month | -7.3384 | -8.2225 | -6.473 | 41.104 | -0.1785 | 0.8583 | -0.1785 |
| Age Subadult | 74.4449 | 64.3651 | 84.2971 | 452.0486 | 0.1647 | 0.8692 | 0.1647 |
| descriptionWoody Wetlands | 6.2264 | 3.9379 | 7.7604 | 43.5394 | 0.143 | 0.8863 | 0.143 |
| knn.dist | 0 | -0.0001 | 0.0001 | 0 | 0.1217 | 0.9031 | 0.1217 |
| Species Red Fox:knn.dist | 0 | 0 | 0 | 0.0003 | -0.0787 | 0.9373 | -0.0787 |
| description Grassland/Herbaceous | -36.4674 | NA | 925.75 | 17730.7986 | -0.0021 | 0.9984 | -0.0021 |
| description Open Water | 17.2418 | -674.18 | NA | 12532.6322 | 0.0014 | 0.9989 | 0.0014 |
| description Scrub/Shrub | 19.7152 | -3537.6 | NA | 17730.3702 | 0.0011 | 0.9991 | 0.0011 |
| description Emergent Herbaceous Wetlands | -18.2004 | NA | 3523.67 | 17730.37 | -0.001 | 0.9992 | -0.001 |

**Table S5. Genbank Accessions and Sequence Data for Canine Morbilliviruses Identified (N=32 Isolates).**

>PQ559088.1 Morbillivirus canis isolate 027A_Raccoon_NC fusion protein (F) and hemagglutinin (H) genes, partial cds

GCACAAKCACAATTATCAATCAGAGTCCTGATAAGTTGCTGACATTTATTGCCTCCGATACCTGTCCACT

GGTTGAAATAGATGGTGTAACTATCCAGGTTGGAGGGAGGCMRKACCCTGATATGGTATACGAAAGCAGA

GTTGCCTTAGGCCCTGCTATATCACTTGAGAGGTTAGATGTAGGGACAAATTTAGGGAACGCCCTTAAGA

AACTGGATGATGCCAAAGTACTGATAGACTCCTCTAACCAGATCCTTGAGACGGTTAGGCGCTCTTCCCT

TAATTTTGGCAGTCTCCTCAGCATTCCTATATTAATCTGTACAGCTCTGGCTTTGTTGTTGCTAATCTAC

TGCTGTAAGAGACGCTACCAACAGACACGCAGGCAGAACCCTAAGGTCGATCCTACATTTAAACCTGATT

TGACTGGAACTTCGAAATCCTATGTAAGATCACTCTGAAGCACTCTGGTCACACGTGTTACCCGATGGTC

AGGCTTGAAATCTATAAATCCCGCCCAATTTCCTTCAAAGGCTATCATACTGCAACAAATTGTGGAGAGG

ACGAACTACGATTCCCGTAATTAAAGAAAACTTAGGGCTCAGGTAGTCCAACAATGCTCTCCTACCGAGA

CAAGGTGGGTGCCTTCTATAAGGACAATGCTAGAGCTAATTTATCCAAGCTGTCATTAGTGACAGAAGAG

CAAGGGGGCAGGAGACCACCCTATTTGCTGTTTGTCCTTCTCATCCTACTGGTTGGAATCATGGCCTTGC

TTGCTATCACTGGAGTTCGATTTCACCAAGTATCAACTAGCAATATGGAATTTAGCAGATTGCTGAAAGA

GGATCTGGAGAAATCAGAGGCCGTACACCACCAAGTCATAGATGTCTTGACGCCGCTCTTCAAAATTATT

GGAGATGAGATTGGGTTACGGTTGCCACAAAAACTAA

>PQ559089.1 Morbillivirus canis isolate 069A_Skunk_MO fusion protein (F) and hemagglutinin (H) genes, partial cds

TCGTCGCAAATTGTGCTTCTATACTGTGTAAGTGTTATAGCACAGGCACAATTATCAATCAGAGTCCTGA

TAAATTGCTGACATTTATTGCATCCGATACCTGCCCATTGGTTGAAATAGATGGTGTAACTATCCAGGTT

GGAGGGAGGCAATATCCTGATATGGTATATGAAAGCAAAGTTGCCTTAGGCCCTGCTATATCACTTGAGA

GGTTAGATGTAGGTACAAATTTAGGGAACGCCCTTAATAAACTGGATGATGCTAAGGTACTGATAGACTC

CTCTAACCAGATCCTTGAGACGGTTAGGCGCTCTTCATTCAATTTTGGCAGTCTTCTCAGTGTTCCCATA

TTAATCTGTACAGCCCTGGCTTTATTGTTGCTGATTTACTGCTGTAAAAGACGCTACCAACAGACACTCA

AGCAGAATACTAAGGTCGATCCGACATTTAAACCTGATTTGACTGGAACTTCGAAATCCTATGTAAGATC

ACTCTGAAGCACCCTGGTCACACGTCTTACCCGATTGTCCGGCTTGAAATATATAAAACCCRCCCAATTT

TCTTCAAAAGCTATCAAACTGCAACAAATAGTGGAGAGGACTGACTACGATTATCGTAATTAAAGAMAAC

TTAGGGCTCAAGTAGTCCGACAATGCTCTCCTACCAAGACAAGGTGGGTGCCTTCTATAAGGATAATGCA

AGAGCTAATTCATCTAGGCTGTCCTTAGTGACAGAAGACCAAGGGGGCAGGAGACCACCCTATTTGCTGT

TTGTCCTTCTCATCCTACTGGTTGGAATCATGGCCTTGCTCGCTATCACTGGAGTTCGATTTCACCAGGT

ATCAACTAGCAATATGGAATTTAGCAGATTGCTGAAAGAGGATATGGAAAAATCAGAGGCCGTACATCAC

CAAGTCATAGATGTCTTGACACCACTCTTCAAAATTATTGGAGATGAGGTTGGGTTACGGTTGCCACAAA

AACTGAACGAGATCAAACAATTTATCCTTCAAAAGACAAACTTCTTTAATCCGAACAGGGAGTTC

>PQ559090.1 Morbillivirus canis isolate 169A_Raccoon_FL fusion protein (F) and hemagglutinin (H) genes, partial cds

ATCGTCGCGAATTGTGCTTCCATACTCTGTAAGTGTTATAGCACAAGCACAATTATCAATCAGAGTCCTG

ATAAGTTGCTGACATTTATTGCCTCCGATACCTGTCCACTGGTTGAAATAGATGGTGTNACTATCCAGGT

TGGAGGGAGGCAGTACCCTGATATGGTATACGAAAGCAGAGTTGCCTTAGGCCCTGCTATATCACTTGAG

AGGTTAGATGTAGGGACAAATTTAGGGAACGCCCTTAAGAAACTGGATGATGCCAAAGTACTGATAGACT

CCTCTAACCAGATCCTTGAGACGGTTAGGCGCTCTTCCCTTAATTTTGGCAGTCTCCTCAGTGTTCCCAT

ATTAATCTGTACAGCTCTGGCTTTGTTGTTGCTAATCTACTGCTATAAGAGACGCTACCAACAGACACTC

AGGCAGAACCCTAAGGTCGATCCTACATTTAAACCTGATTTGACTGGAACTTCGAAATCCTATGTAAGAT

CACTCTGAAGCACTCTGGTCACACGTGTTACCCGATGGTCAGGCTTGAAATCTATAAATCCCGCCCAATT

CCCTTCAAAGGCTATCAAACTGCAACAAAATGTGAAGAGGACGAACTACGATTCTCGTAATTAAAGAAAA

CTTAGGGCTCAGGTAGTCCAACAATGCTCTCCTACCGAGACAAGGTGGGTGCCTTCTATAAGGACAATGC

TAGAGCTAATTCATCCAAGCTGTCCTTAGTGACAGAAGAGCAAGGGGGCAGGAGACCACCCTATTTGCTG

TTTGTCCTTCTCATCCTACTGGTTGGAATCATGGCCTTGCTTGCTATCACTGGAGTTCGATTTCACCAAG

TATCAACTAGCAATATGGAATTTAGCAGATTGCTGAAAGAGGATCTGGAGAAATCAGAGGCCGTACATCA

CCAAGTCATAGATGTCTTGACGCCGCTCTTCAAAATTATTGGGGATGAGGTTGGGTTACGGTTGCCACAA

AAACTAAACGAGATCAAACAATTTATCCTTCAAAAGACAAACTTCTTCAATCCGAACAGGGAATTC

>PQ559091.1 Morbillivirus canis isolate 317A_Gray_Fox_NC fusion protein (F) and hemagglutinin (H) genes, partial cds

ATCGTCGCGAATTGTGCTTCTATACTCTGTAAGTGTTATAGCACAAGCACAATTATCAATCAGAGTCCTG

ATAAGTTGCTGACATTTATTGCCTCCGATACCTGTCCACTGGTTGAAATAGATGGTGTAACTATCCAGGT

TGGAGGGAGGCAGTACCCTGATGTGGTATACGAAAGCAGAGTTGCCTTAGGCCCTGCTATATCACTTGAG

AGGTTAGATGTAGGGACAAATTTAGGGAACGCCCTTAAGAAACTGGATGATGCCAAAGTACTGATAGACT

CCTCTAACCAGATCCTTGAGACGGTTAGGCGCTCTTCCCTTAATTTTGGCAGTCTCCTCAGCGTTCCCAT

ATTAATCTGTACAGCTCTGGCTTTGTTGTTGCTAATCTACTGCTGTAAGAGACGCTACCAACAGACACTC

AGGCAGAACCCTAAGGTCGATCCTACATTTAAACCTGATTTGACTGGAACTTCGAAATCCTATGTAAGAT

CACTCTGAAGCACTCTGGTCACACGTGTTACCCGATGGTCAGGCTTGAAATCTATAAATCCCGCCCAATT

TCCCTCAAAGGCTATCAAACTGCAACAAATTGCGGAGAGGACGAACTACGATTCCCGTAATTAAAGAAAA

CTTAGGGCTCAGGTAGTCCAACAATGCTCTCCTACCGAGACAAGGTGGGTGCCTTCTATAAGGACAATGC

TAGAGCTAATTTATCCAAGCTGTCCTTAGTGACAGAAGAGCAAGGGGGCAGGAGACCACCCTATTTACTG

TTTGTCCTTCTCATCCTACTGGTTGGAATCATGGCCTTGCTTGCTATCACAGGAGTTCGATTTCACCAAG

TATCAACTAGCAATATGGAATTTAGCAGATTGCTGAAAGAGGATCTGGAGAAATCAGAGGCCGTACATCA

CCAAGTCATAGATGTCTTGACGCCGCTCTTCAAAATTATTGGAGATGAGATTGGGTTACGGTTGCCACAA

AAACTAAACGAGATCAAACAATTTATCCTTCAAAAAACAAACTTCTTCAATCCGAACAGGGAATTC

>PQ559092.1 Morbillivirus canis isolate 383A_Gray_Fox_AR fusion protein (F) and hemagglutinin (H) genes, partial cds

TTGTGCTTCCATACTGTGTAAGTGTTATAGCACAGGCACAATTATCAATCAAAGTCCTGATAAATTGCTG

ACATTTATTGCCTCTGATACTTGCCCGCTGGTTGAAATAGATGGTGTAACTATCCAGGTTGGAGGGAGGC

AATACCCTGATATGGTATACGAAAGCAAAGTCGCCTTGGGCCCTGCTATATCACTCGAGAGGTTAGATGT

AGGTACAAATTTAGGGAACGCTCTCAAGAAACTAGATGAGGCTAAGGTACTGATAGACTCTTCTAACCAG

ATCCTTGAGACGGTTAGGCGCTCTTCCTTTAATTTTGGCAGTCTTCTTGGTGTTCCCATATTAATCTGTA

CAGCCCTGGCTTTATTGTTGCTGATTTACTGCTGTAAAAGACGCTACCAACAGACACTCAAGCAGAATAC

TAAGGTCGATCCGACATTTAAACCTGATTTGACTGGAACCTCGAAATCCTATGTAAGATCACTCTGAGGC

ACTCTGCTCACACGTCTTACCCGATTGTCAGGCTTGAAATATATAAATTCCGCCCAATTTTCCTCAAAAG

CTATCAAACTGCAACAAATAGTGGAGAGGTCTGACTACGATTATCGTAATTAAAGAAAACTTAGGGCTCA

GGTAGTCCAACAATGCTCTCCTACCAAGACAAGGTGGGTGCCTTCTATAAGGACAATGCAAGAGCCAATT

CATCTAGGCTGTTCTTAGTGACAGAAGACCAAGGGGGCAGGAGACCACCCTATTTGCTGTTTGTCCTGCT

CATCCTACTGGTTGGAATCATGGCCTTGCTCGCTATTACTGGAGTTCGATTTCACCAAGTATCAACTAGC

AATATGGAATTTAGCAGATTGCTGAAAGAGGATATGGAGAAATCAGAGGCCGTACATCACCAAGTCATAG

ATGTCTTGACACCACTCTTCAAAATTATTGGAGATGAGATTGGGTTACGGTTGCCACAAAAACTGAACGA

GATCAAACAATTTATCCTTCAAAAGACAAACTTCTTTAATCCGAACAGGGAGTTC

>PQ559093.1 Morbillivirus canis isolate 408A_Gray_Fox_NC fusion protein (F) and hemagglutinin (H) genes, partial cds

ATCGTCGCGAATTGTGCTTCTATACTCTGTAAGTGTTATAGCACAAGCACAATTATCAATCAGAGTCCTG

ATAAGTTGCTGACATTTATTGCCTCCGATACCTGTCCACTGGTTGAAATAGATGGTGTAACTATCCAGGT

TGGAGGGAGGCAGTACCCTGATGTGGTATACGAAAGCAGAGTTGCCTTAGGCCCTGCTATATCACTTGAG

AGGTTAGATGTAGGGACAAATTTAGGGAACGCCCTTAAGAAACTGGATGATGCCAAAGTACTGATAGACT

CCTCTAACCAGATCCTTGAGACGGTTAGGCGCTCTTCCCTTAATTTTGGCAGTCTCCTCAGCGTTCCCAT

ATTAATCTGTACAGCTCTGGCTTTGTTGTTGCTAATCTACTGCTGTAAGAGACGCTACCAACAGACACTC

AGGCAGAACCCTAAGGTCGATCCTACATTTAAACCTGATTTGACTGGAACTTCGAAATCCTATGTAAGAT

CACTCTGAAGCACTCTGGTCACACGTGTTACCCGATGGTCAGGCTTGAAATCTATAAATCCCGCCCAATT

CCCCTCAAAGGCTATCAAACTGCAGCAAATTGCGGAGAGGACGAACTACGATTCCCGTAATTAAAGAAAA

CTTAGGGCTCAGGTAGTCCAACAATGCTCTCCTACCGAGACAGGGTGGGTGCCTTCTATAAGGACAATGC

TAGAGCTAATTTATCCAAGCTGTCCTTAGTGACAGAAGAGCAAGGGGGCAGGAGACCACCCTATTTACTG

TTTGTCCTTCTCATCCTACTGGTTGGAATCATGGCCTTGCTTGCTATCACAGGAGTTCGATTTCACCAAG

TATCAACTAGCAATATGGAATTTAGCAGATTGCTGAAAGAGGATCTGGAGAAATCAGAGGCCGTACATCA

CCAAGTCATAGATGTCTTGACGCCGCTCTTCAAAATTATTGGAGATGAGATTGGGTTACGGTTGCCACAA

AAACTAAACGAGATCAAACAATTTATCCTTCAAAAAACAAACTTCTTCAATCCGAACAGGGAATTC

>PQ559094.1 Morbillivirus canis isolate 439A_Skunk_MO fusion protein (F) and hemagglutinin (H) genes, partial cds

TCGTCGCAAATTGTGCTTCTATACTGTGTAAGTGTTATAGCACAGGCACAATTATCAATCAAAGTCCTGA

TAAATTGCTGACATTTATTGCCTCCGATACTTGCCCACTGGTTGAAATAGATGGTGTAACTATCCAGGTT

GGAGGGAGGCAATACCCTGATATGGTATACGAAAGCAAAGTTGCCTTAGGCCCTGCTATATCACTTGAGA

GGTTAGATGTAGGTACAAATTTAGGGAACGCCCTTAAGAAACTAGATAATGCTAAGGTACTGATAGACTC

CTCTAACCAGATCCTTGAGACGGTTAGGCGCTCTTCCTTTAATTTTGGCAGTCTTCTCAGTGTTCCCATA

TTAATCTGTACAGCCCTGGCCTTATTGTTGCTGATTTACTGTTGTAAAAGACGCTACCGACAGACACTCA

AGCAGAATACCAAAGTCGATCCGACATTTAAACCTGATTTGACTGGAACTTCGAAATCCTATGTAAGATC

ACTCTGAGGCACTCTGGTCACACGTCTTACCCGATTGTCAGGCTTAAAATATACAAATCCCGCCCAATCC

TTCTCAAAAGCTATCAAACTGCAACAAATAGTAGAGAGGATTGACCACAACCATCGTAATTAAAGAAAAC

TTAGGGCTCAGGTAGTCCAACAATGCTCTCCTACCACGACAAGGTGGGTGCCTTCTATAAGGATAATGCA

AGAGCTAATTCATCTAGGCTGTCCTTAGTGACAGAAGACCAAGGGGGCAGGAGACCGCCCTATTTGCTGT

TTGTGCTTCTCATCCTACTGGTTGGAATCATGGCCTTGCTCGCTATCACTGGAGTTCGATTTCACCAAGT

ATCAACTAGCAATATGGAATTTAGCAGATTGCTGAAAGAGGATATGGAGAAATCAGAGGCCGTACATCAC

CAAGTCATAGATGTCTTGACACCACTCTTCAAAATTATTGGAGATGAGATTGGGTTACGGTTGCCACAAA

AACTGAACGAGATCAAACAATTTATCCTTCAAAAGACAAACTTCTTCAATCCGAAGAGGGAGTTC

>PQ559095.1 Morbillivirus canis isolate 440A_Skunk_MO fusion protein (F) and hemagglutinin (H) genes, partial cds

TCGTCGCAAATTGTGCTTCTATACTGTGTAAGTGTTATAGCACAGGCACAATTATCAATCAAAGTCCTGA

TAAATTGCTGACATTTATTGCCTCCGATACTTGCCCACTGGTTGAAATAGATGGTGTAACTATCCAGGTT

GGAGGGAGGCAATACCCTGATATGGTATACGAAAGCAAAGTTGCCTTAGGCCCTGCTATATCACTTGAGA

GGTTAGATGTAGGTACAAATTTAGGGAACGCCCTTAAGAAACTAGATAATGCTAAGGTACTGATAGACTC

CTCTAACCAGATCCTTGAGACGGTTAGGCGCTCTTCCTTTAATTTTGGCAGTCTTCTCAGTGTTCCCATA

TTAATCTGTACAGCCCTGGCCTTATTGTTGCTGATTTACTGTTGTAAAAGACGCTACCGACAGACACTCA

AGCAGAATACCAAAGTCGATCCGACATTTAAACCTGATTTGACTGGAACTTCGAAATCCTATGTAAGATC

ACTCTGAGGCACTCTGGTCACACGTCTTACCCGATTGTCAGGCTTAAAATATACAAATCCCGCCCAATCC

TTCTCAAAAGCTATCAAACTGCAACAAATAGTAGAGAGGATTGACCACGACCATCGTAATTAAAGAAAAC

TTAGGGCTCAGGTAGTCCAACAATGCTCTCCTACCACGACAAGGTGGGTGCCTTCTATAAGGATAATGCA

AGAGCTAATTCATCTAGGCTGTCCTTAGTGACAGAAGACCAAGGGGGCAGGAGACCGCCCTATTTGCTGT

TTGTGCTTCTCATCCTACTGGTTGGAATCATGGCCTTGCTCGCTATCACTGGAGTTCGATTTCACCAAGT

ATCAACTAGCAATATGGAATTTAGCAGATTGCTGAAAGAGGATATGGAGAAATCAGAGGCCGTACATCAC

CAAGTCATAGATGTCTTGACACCACTCTTCAAAATTATTGGAGATGAGATTGGGTTACGGTTGCCACAAA

AACTGAACGAGATCAAACAATTTATCCTTCAAAAGACAAACTTCTTCAATCCGAAGAGGGAGTTC

>PQ559096.1 Morbillivirus canis isolate 575A_Raccoon_NC fusion protein (F) and hemagglutinin (H) genes, partial cds

ATCGTCGCGAATTGTGCTTCTATACTCTGTAAGTGTTATAGCACAAGCACAATTATCAATCAGAGTCCTG

ATAAGTTGCTGACATTTATTGCCTCCGATACCTGTCCACTGGTTGAAATAGATGGTGTAACTATCCAGGT

TGGAGGGAGGCAGTACCCTGATATGGTATACGAAAGCAGAGTTGCCTTAGGCCCTGCTATATCACTTGAG

AGGTTAGATGTAGGGACAAATTTATGGAACGCCCTTAAGAAACTGGATGATGCCAAAGTACTGATAGACT

CCTCTAACCAGATCCTTGAGACGGTTAGGCGCTCTTCCCTTAATTTTGGCAGTCTCCTCAGCATTCCTAT

ATTAATCTGTACAGCTCTGGCTTTGTTGTTGCTAATCTACTGCTGTAAGAGACGCTACCAACAGACACGC

AGGCAGAACCCTAAGGTCGATCCTACATTTAAACCTGATTTGACTGGAACTTCGAAATCCTATGTAAGAT

CACTCTGAAGCACTCTGGTCACACGTGTTACCCGATGGTCAGGCTTGAAATCTATAAATCCCGCCCAATT

TCCTTCAAAGGCTATCATACTGCAACAAATTGTGGAGAGGACGAACTACGATTCCCGTAATTAAAGAAAA

CTTAGGGCTCAGGTAGTCCAACAATGCTCTCCTACCGAGACAAGGTGGGTGCCTTCTATAAGGACAATGC

TAGAGCTAATTTATCCAAGCTGTCATTAGTGACAGAAGAGCAAGGGGGCAGGAGACCACCCTATTTGCTG

TTTGTCCTTCTCATCCTACTGGTTGGAATCATGGCCTTGCTTGCTATCACTGGAGTTCGATTTCACCAAG

TATCAACTAGCAATATGGAATTTAGCAGATTGCTGAAAGAGGATCTGGAGAAATCAGAGGCCGTACACCA

CCAAGTCATAGATGTCTTGACGCCGCTCTTCAAAATTATTGGAGATGAGATTGGGTTACGGTTGCCACAA

AAACTAAACGAGATTAAACAATTTATCCTTCAAAAGACAAACTTCTTCAATCCGAACAGGGAATTC

>PQ559097.1 Morbillivirus canis isolate 62A_Gray_Fox_GA fusion protein (F) and hemagglutinin (H) genes, partial cds

ATCGTCGCGAATTGTGCTTCTATACTCTGTAAGTGTTATAGCACAAGCACAATTATCAATCAGAGTCCTG

ATAAGTTGCTGACATTTATTGCCTCCGATACCTGTCCACTGGTTGAAATAGATGGTGTAACTATCCAGGT

TGGAGGGAGGCAGTACCCTGATATGGTATACGAAAGCAAAGTTGCCTTAGGCCCTGCTATATCACTTGAG

AGGTTAGATGTAGGGACAAATTTAGGGAACGCCCTTAGGAAACTGGACGATGCCAAAGTACTGATAGACT

CCTCCAACCAGATCCTTGAGACGGTTAGGCGCTCTTCCCTTAATTTTGGCAGTCTCCTCAGCGTTCCCAT

ATTAATCTGTACAGCTCTGGCTTTGTTGTTGCTAATCTACTGCTGTAAGAGACGCTACCAACAGACACTC

AGGCAGAACCCTAAGGTCGATCCTACATTTAAACCTGATTTGACTGGAACGTCGAAATCCTATGTAAGAT

CACTCTGAAGCACTCTGGTCACACGTGTTACCCGATGGTCAGGCTTGAAATCTATAAATCCCGCCCAATT

TCCTTCAAAGGCCATCAAACTGCAACAAATTGTGGAGAGGACGAACTACGATTCTCGTAATTAAAGAAAA

CTTAGGGCTCAAGTAGTCCAACAATGCTCTCCTACCGAGACAAGGTGGGTGCCTTCTATAAGGACAATGC

TAGAGCTAATTCATCCAAGTTGTCCTTAGTGACAGAAGAGCAAGGGGGCAGGAGACCACCCTATTTGCTG

TTTGTCCTTCTCATCCTACTGGTTGGAATCATGGCCTTGCTTGCTATCACTGGAGTTCGATTTCACCAAG

TATCAACTAGCAATATGGAGTTTAGCAGATTGCTGAAAGAGGATCTGGAGAAATCAGAGGCCGTACATCA

CCAAGTCATAGATGTCTTGACGCCGCTCTTCAAAATTATTGGAGATGAGATTGGGTTACGGTTGCCACAA

AAACTAAACGAGATCAAACAATTTATCCTTCAAAAGACAAACTTCTTCAATCCGAACAGGGAATTC

>PQ559098.1 Morbillivirus canis isolate 776A_Raccoon_GA fusion protein (F) and hemagglutinin (H) genes, partial cds

TTGTGCTTCTATACTGTGTAAGTGTTATAGCACAGGCACAATCATCAATCAAAGTCCTGATAAATTGCTG

ACATTTATTGCCTCCGATACCTGCCCACTGGTTGAAATAGATGGTGTAACTATCCAGGTTGGAGGGAGGC

AATACCCTGATATGGTATACGAAAGTAAAGTTGCCTTAGGCCCTGCTATATCGCTTGAGAGGTTAGATGT

AGGTACAAATTTAGGGAACGCCCTTAAGAAACTGGATGATGCTAAGGTATTGATAGACTCCTCTAACCAG

GTCCTTGAGACGGTTAGGCGCTCTTCATTTAATTTTGGCAGTCTTCTCAGTGTTCCCATATTAATCTGTA

CAGCCCTGGCTTTATTGTTGCTGATTTACTGCTGTAAAAGACGCTACCAACAGACACTCAAGCAGAATAC

TAAGGTCGACCCGACATTTAAACCTGATTTGACTGGGACTTCGAAATCCTATGTAAGATCACTCTGAAGC

ACTCTAATCACACGCCTTACCCGATTGTCAGGCTTGAAATATATAAGTTCCGCCCAATTTTCTTCAAAAG

CTATCAAACTACAACAAATAGTGGAGAGGACTGACTACGATTATCGTAATTAAAGAAAACTTAGGGCTCA

GGTAGTCCAACAATGCTCTCCTACCAAGACAAGGTGGGTGCCTTCTATAAGGATAATGCAAGAGCTAATT

CATCTAGGCTGTCCTTAGTGACAGAAGACCAAGGGGGCAGGAGACCACCCTATTTGCTGTTTGTCCTTCT

CATCCTACTGGTTGGAATCATGGCCTTGCTCGCTATCACTGGAGTTCGATTTCACCAAGTATCAACTAGT

AATATGGAATTTAGCAGATTGCTGAAAGAGGATATGGAGAAATCAGAGGCCGTACATCACCAAGTCATAG

ATGTCTTGACACCACTCTTCAAAATTATTGGAGATGAGATTGGGTTACGGTTGCCACAAAAACTGAACGA

GATCAAACAATTTATCCTTCNAAAGACAAACTTCTTTAATCCGAACAGGGAGTTC

>PQ559099.1 Morbillivirus canis isolate 819A_Gray_Fox_NC fusion protein (F) and hemagglutinin (H) genes, partial cds

TCGTCGCGAATTGTGCTNCTATACTCTGTAAGTGTTATAGCACAAGCACAATTATCAATCAGAGTCCTGA

TAAGTTGCTGACATTTATTGCCTCCGATACCTGTCCACTGGTTGAAATAGATGGTGTAACTATCCAGGTT

GGAGGGAGGCAGTACCCTGATGTGGTATACGAAAGCAGAGTTGCCTTAGGCCCTGCTATCTCACTTGAGA

GGTTAGATGTAGGGACAAATTTAGGGAACGCCCTTAAGAAACTGGATGATGCCAAAGTACTGATAGACTC

CTCTAACCAGATCCTTGAGACGGTTAGGCGCTCTTCCCTTAATTTTGGCAGTCTCCTCAGCGTTCCCATA

TTAATCTGTACAGCTCTGGCTTTGTTGTTGCTAATCTACTGCTGTAAGAGACGCTACCAACAGACACTCA

GGCAGAACCCTAAGGTCGATCCTACATTTAAACCTGATTTGACTGGAACTTCGAAATCCTATGTAAGATC

ACTCTGAAGCACTCTGGTCACACGTGTTACCCGATGGTCAGGCTTGAAATCTATAAATCCCGCCCAATTT

CCCTCAAAGGCTATCAAACTGCAACAAATTGCGGAGAGGACGAACTACGATTCCCGTAATTAAAGARAAC

TTAGGGCTCAGGTAGTCCAACAATGCTCTCCTACCGAGACAAGGTGGGTGCCTTCTATAAGGACAATGCT

AGAGCTAATTTATCCAAGCTGTCCTTAGTGACAGAAGAGCAAGGGGGCAGGAGACCACCCTATTTACTGT

TTGTCCTTCTCATCCTACTGGTTGGAATCATGGCCTTGCTTGCTATCACAGGAGTTCGATTTCACCAAGT

ATCAACTAGCAATATGGAATTTAGCAGATTGCTGACAGAGGATCTGGAGAAATCAGAGGCCGTACATCAC

CAAGTCATAGATGTCTTGACGCCGCTCTTCAAAATTATTGGAGATGAGATTGGGTTACGGTTGCCACAAA

AACTAAACGAGATCAAACAATTTATCCTTCAAAAAACAAACTTCTTCAATCCGAACAGGGAATTC

>PQ559100.1 Morbillivirus canis isolate 900A_Raccoon_NC fusion protein (F) and hemagglutinin (H) genes, partial cds

ATCGTCGCGAATTGTGCTTCTATACTCTGTAAGTGTTATAGCACAAGCACAATTATCAATCAGAGTCCTG

ATAAGTTGCTGACATTTATTGCCTCCGATACCTGTCCACTGGTTGAAATAGATGGTGTAACTATCCAGGT

TGGAGGGAGGCATTACCCTGATATGGTATACGAAAGCAGAGTTGCCTTAGGCCCTGCTATATCACTTGAG

AGGTTAGATGTAGGGACAAATTTAGGGAACGCCCTTAAGAAACTGGATGATGCCAAAGTACTGATAGACT

CCTCTAACCAGATCCTTGAGACGGTTAGGCGCTCTTCCCTTAATTTTGGCAGTCTCCTCAGCGTTCCCAT

ATTAATCTGTACAGCTCTGGCTTTGTTGTTGCTAATCTACTGCTGTAAGAGACGCTACCAACAGACACTC

AGGCAGAACCCTAAGGTCGATCCTACATTTAAACCTGATTTGACTGGAACTTCGAAATCATATGTAAGAT

CACTCTGAAGCACTCTGGTCACACGTGTTACCCGATGATCAGGCTTGAAATCTATAAAGCCCGCCCAATT

TCCTTCAAAGGCTATCAAACTGCAACAAATTGTGGAGAGGACGAACTACGATTCCCGTAATTAAAGARAA

CTTAGGGCTCAGGTAGTCCAACAATGCTCTCCTACCGAGACAAGGTGGGTGCCTTCTATAAGGACAATGC

TAGAGCTAATTTATCCAAGCTGTCCTTAGTGACAGAAGAGCAAGGGAGCAGGAGACCACCCTATTTGCTG

TTTGTCCTTCTCATCCTACTGGTTGGAATCATGGCCTTGCTTGCTATCACTGGAGTTCGATTTCACCAAG

TATCAACTAGCAATATGGAATTTAGCAGATTGCTGAAAGAGGATCTGGAGAAATCAGAGGCCGTACATCA

CCAAGTCATAGATGTCTTGACGCCGCTCTTCAAAATTATCGGAGATGAGATTGGGTTACGGTTGCCACAA

AAACTAAACGAGATCAAACAATTTATCCTTCAAAAAACAAACTTCTTCAATCCGAACAGGGAATTC

>PQ559101.1 Morbillivirus canis isolate 940A_Raccoon_NC fusion protein (F) and hemagglutinin (H) genes, partial cds

CCAATCARAGTCCTGATAAGTTGCTGACATTTATTGCCTCCGATACTTGTCCACTGGTTGAAATAGATGG

TGTAACTATCCAGGTTGGAGGGAGGCAGTACCCAGATGTGGTATACGAAAGCAGAGTTGCCTTAGGCCCT

GCTATATCACTTGAGAGGTTAGATGTAGGGACAAATTTAGGGAACGCCCTTAAGAAACTGGATGATGCCA

AAGTACTGATAGACTCCTCTAACCAGATCCTTGAGACGGTTAGGCGCTCTTCCCTTAATTTTGGCAGTCT

CCTCAGCGTTCCCATATTAATCTGTACAGCTCTGGCTTTGTTGTTGCTAATCTACTGCTGTAAGAGACGC

TACCAACAGACACTCAGGCAGAACCCTAAGGTCGATCCTACATTTAAACCTGATTTGACTGGAACTTCGA

AATCCTATGTAAGATCACTCTGAAACACTCTGGTCACACGTGTTACCCGATGGTCAGGCTTGAAATCTAT

AAATCCCGCCCAATTTCCCTCAAAGGCTATCAAACTGCAACAAATTGCGGAGAGGACGAACTACGATTCC

CGTAATTAAAGAAAACTTAGGGCTCAGGTAGTCCAACAATGCTCTCCTACCGAGACAAGGTGGGTGCCTT

CTATAAGGACAATGCTAGAGCTAATTTATCCAAGCTGTCCTTAGTGACAGAAGAGCAAGGGGGCAGGAGA

CCACCCTATTTTCTGTTTGTCCTTCTCATCCTACTGGTTGGAATCATGGCCTTGCTTGCTATCACAGGAG

TTCGATTTCACCAAGTATCAACTAGCAATATGGAATTTAGCAGATTGCTGAAAGAGGATCTGGAGAAATC

AGAGGCCGTACATCACCAAGTCATAGATGTCTTGACGCCGCTCTTCAAAATTATTGGAGATGAGATTGGG

TTACGGTTGCCACAAAAACTAAACGAGATCAAACAATTTATCCTTCAAAAAACAAACTTCTTCAATCCGA

ACAGGGAATTC

>PQ559102.1 Morbillivirus canis isolate 973A_Raccoon_FL fusion protein (F) and hemagglutinin (H) genes, partial cds

ATCGTCGCGAATTGTGCTTCTATACTCTGTAAGTGTTATAGCACAAGCACAATTATCAATCAGAGTCCTG

ATAAGTTGCTGACATTTATTGCCTCCGATACCTGTCCACTGGTTGAAATAGATGGTGTAACTATCCAGGT

TGGAGGGAGACAGTACCCTGATATGGTATACGAAAGCAAAGTTGCCTTAGGCCCTGCTATATCACTTGAG

AGGTTAGATGTAGGGACAAATTTAGGAAACGCCCTTAGGAAACTGGATGATGCCAAAGTACTGATAGACT

CCTCTAACCAGATCCTTGAGACGGTTAGGCGCTCTTCCCTTAATTTTGGCAGTCTCCTTAGCGTTCCCAT

ATTAATCTGTACAGCTCTGGCTTTGTTGTTGCTAATCTACTGCTGTAAGAGACGCTACCAACAGACACTC

AGGCAGAACCCTAAGGTCGATCCTACATTTAAACCTGATTTGACTGGAACGTCGAAATCCTATGTAAGAT

CACTCTGAAGCACTCTGGTCACACGTGTTACCCGATGGTCAGGCTTGAAATCTATAATTCCCGCCCAATT

TCCTTCAAAGGCTATCAAACTGCAACAAATTGTGGAGAGGACGAACTACGATTCTCGTAATTAAAGAAAA

CTTAGGGCTCAAGTAGTCCAACAATGCTCTCCTACCGAGACAAGGTGGGTGCCTTCTATAAGGACAATGC

TAGAGCTAATTCATCCAAGTTGTCCTTAGTGACAGAAGAGCAAGGGGGCAGGAGACCACCCTATTTGCTG

TTTGTCCTTCTCATCCTACTGGTTGGAATCATGGCCTTGCTTGCTATCACTGGAGTTCGATTTCACCAAG

TATCAACTAGCAATATGGAGTTTAGCAGATTGCTGAAAGAGGATCTGGAGAAATCAGAGGCCGTACATCA

CCAAGTCATAGATGTCTTGACGCCGCTCTTCAAAATTATTGGAGATGAGATTGGGTTACGGTTGCCACAA

AAACTAAACGAGATCAAACAATTTATCCTTCAAAAGACAAACTTCTTCAATCCGAACAGGGAATTC

>PQ559103.1 Morbillivirus canis isolate 979A_Raccoon_MO fusion protein (F) and hemagglutinin (H) genes, partial cds

TTGTGCCTCTATACTATGTAAGTGTTATAGCACAGGCACAATTATCAATCAAAGTCCTGATAAATTGCTG

ACATTTATTGCCTCCGATACCTGCCCACTGGTTGAAATAGATGGTGTAACTATCCAGGTTGGAGGGAGGC

AATACCCTGATATGGTATACGAAAGCAAAGTCGCCTTGGGCCCTGCTATATCACTTGAGAGGTTAGATGT

AGGTACAAATTTAGGGAACGCTCTTAAGAAACTAGATGAGGCTAAGGTACTGATAGACTCCTCTAACCAG

ATCCTTGAAACGGTTAGGCGCTCTTCCTTTAATTTTGGCAGCCTTCTTAGTGTTCCCATATTAATCTGTA

CAGCCCTGGCTTTATTGTTGCTGATTTACTGCTGTAAAAGACGCTACCAACAGACACTCAAGCAGAATAC

TAAGGTCGATCCGACATTTAAACATGATTTGACTGGAACTTCGAAATCCTATGTAAGATCACTCTGAGGC

ACTCTGCTCACACGTCTTACTCGATTGTCAGGCTTGAAATATATAAATTCCGCCCAATTTTCTTCAAAAG

CTATCAAACTGCAACAAATAGTGGAGAGGTCTGACTACGATTATCGTAATTAAAGAAAACTTAGGGCTCA

GGTAGTCCAACAATGCTCTCCTACCAAGACAAGGTGGGTGCCTTCTATAAGGACAATGCAAGAGCCAATT

CATCTAGGCTGTCCTTAGTGACGGAAGAACAAGGGGGCAGGAGACCACCCTATTTGCTGTTTGTCCTGCT

CATCCTACTGGTTGGAATCATGGCCTTGCTCGCTATTACTGGAGTTCGATTTCACCAAGTGTCAACTAGC

AATATGGAATTTAGCAGATTGCTGAAAGAGGATATGGAGAAATCAGAGGCCGTACATCACCAAGTCATAG

ATGTCTTGACACCACTCTTCAAAATTATTGGAGATGAGATTGGGTTACGGTTGCCACAAAAACTGAACGA

GATCAAACAATTTATCCTTCAAAAGACAAACTTCTTTAATCCGAACAGGGAGTTC

>PQ559104.1 Morbillivirus canis isolate A116A_Raccoon_NC fusion protein (F) and hemagglutinin (H) genes, partial cds

ATCGTCGCGAATTGTGCTTCTATACTCTGTAAGTGTTATAGCACAAGCACAATTATCAATCAGAGTCCTG

ATAAGTTGCTGACATTTATTGCCTCCGATACCTGTCCACTGGTTGAAATAGATGGTGTAACTATCCAGGT

TGGAGGGAGGCAGTACCCTGATGTGGTATACGAAAGCAGAGTTGCCTTAGGCCCTGCTATATCACTTGAG

AGGTTAGATGTAGGGACAAATTTAGGGAACGCCCTTAAGAAACTGGATGATGCCAAAGTACTGATAGACT

CCTCTAACCAGATCCTTGAGACGGTTAGGCGCTCTTCCCTTAATTTTGGCAGTCTCCTCAGCGTTCCCAT

ATTAATCTGTACAGCTCTGGCTTTGTTGTTGCTAATCTACTGCTGTAAGAGACGCTACCAACAAACACTC

AGGCAGAACCCTAAGGTCGATCCTACATTTAAACCTGATTTGACTGGAACTTCGAAATCCTATGTAAGAT

CACTCTGAAGCACTCTGGTCACACGTGTTACCCGATGGTCAGGCTTGAAATCTATAAATCCCGCCCAATT

TCCCTCAAAGGCTATCAAACTGCAACAAATTGCGGAGAGGACGAACTACGATTCCCGTAATTAAAGAAAA

CTTAGGGCTCAGGTAGTCCAACAATGCTCTCCTACCGAGACAAGGTGGGTGCCTTCTATAAGGACAATGC

TAGAGCTAATTTATCCAAGCTGTCCTTAGTGACAGAAGAGCAAGGGGGCAGGAGACCACCCTATTTACTG

TTTGTCCTTCTCATCCTACTGGTTGGAATCATGGCCTTGCTTGCTATCACAGGAGTTCGATTTCACCAAG

TATCAACTAGCAATATGGAATTTAGCAGATTGCTGAAAGAGGATCTGGAGAAATCAGAGGCCGTACATCA

CCAAGTCATAGATGTCTTGACGCCGCTCTTCAAAATTATTGGAGATGAGATTGGGTTACGGTTGCCACAA

AAGTTAAACGAGATCAAACAATTTATCCTTCAAAAAACAAACTTCTTCAATCCGAACAGGGAATTC

>PQ559105.1 Morbillivirus canis isolate B116A_Raccoon_NC fusion protein (F) and hemagglutinin (H) genes, partial cds

ATCGTCGCGAATTGTGCTTCTATACTCTGTAAGTGTTATAGCACAAGCACAATTATCAATCAGAGTCCTG

ATAAGTTGCTGACATTTATTGCCTCCGATACCTGTCCACTGGTTGAAATAGATGGTGTAACTATCCAGGT

TGGAGGGAGGCAGTACCCTGATGTGGTATACGAAAGCAGAGTTGCCTTAGGCCCTGCTATATCACTTGAG

AGGTTAGATGTAGGGACAAATTTAGGGAACGCCCTTAAGAAACTGGATGATGCCAAAGTACTGATAGACT

CCTCTAACCAGATCCTTGAGACGGTTAGGCGCTCTTCCCTTAATTTTGGCAGTCTCCTCAGCGTTCCCAT

ATTAATCTGTACAGCTCTGGCTTTGTTGTTGCTAATCTACTGCTGTAAGAGACGCTACCAACAAACACTC

AGGCAGAACCCTAAGGTCGATCCTACATTTAAACCTGATTTGACTGGAACTTCGAAATCCTATGTAAGAT

CACTCTGAAGCACTCTGGTCACACGTGTTACCCGATGGTCAGGCTTGAAATCTATAAATCCCGCCCAATT

TCCCTCAAAGGCTATCAAACTGCAACAAATTGCGGAGAGGACGAACTACGATTCCCGTAATTAAAGAAAA

CTTAGGGCTCAGGTAGTCCAACAATGCTCTCCTACCGAGACAAGGTGGGTGCCTTCTATAAGGACAATGC

TAGAGCTAATTTATCCAAGCTGTCCTTAGTGACAGAAGAGCAAGGGGGCAGGAGACCACCCTATTTACTG

TTTGTCCTTCTCATCCTACTGGTTGGAATCATGGCCTTGCTTGCTATCACAGGAGTTCGATTTCACCAAG

TATCAACTAGCAATATGGAATTTAGCAGATTGCTGAAAGAGGATCTGGAGAAATCAGAGGCCGTACATCA

CCAAGTCATAGATGTCTTGACGCCGCTCTTCAAAATTATTGGAGATGAGATTGGGTTACGGTTGCCACAA

AAGTTAAACGAGATCAAACAATTTATCCTTCAAAAAACAAACTTCTTCAATCCGAACAGGGAATTC

>PQ559106.1 Morbillivirus canis isolate W004A_Raccoon_MO fusion protein (F) and hemagglutinin (H) genes, partial cds

GTCGCAAATTGTGCTTCCATACTGTGTAAGTGTTATAGCACAGGCACAATTATCAATCAAAGTCCTGATA

AATTGCTGACATTTATTGCCTCTGATACTTGCCCGCTGGTTGAAATAGATGGTGTAACTATCCAGGTTGG

AGGGAGGCAATACCCTGATATGGTATACGAAAGCAAAGTCGCCTTGGGCCCTGCTATATCACTTGAGAGG

TTAGATGTAGGTACAAATTTAGGGAACGCTCTCAAGAAACTAGATGAGGCTAAGGTACTGATAGACTCTT

CTAACCAGATCCTTGAGACGGTTAGGCGCTCTTCCTTTAATTTTGGCAGTCTTCTTGGTGTTCCCATATT

AATCTGTACAGCCCTGGCTTTATTGTTGCTGATTTACTGCTGTAAAAGACGCTACCAACAGACACTCAAG

CAGAATACTAAGGTCGATCCGACATTTAAACCTGATTTGACTGGAACCTCGAAATCCTATGTAAGATCAC

TCTGAGGCACTCTGCTCACACGTCTTACCCGATTGTCAGGCTTGAAATATATAAATTCCGCCCAATTTTC

CTCAAAAGCTATCAAACTGCAACAAATAGTGGAGAGGTCTGACTACGATTATCGTAATTAAAGAAAACTT

AGGGCTCAGGTAGTCCAACAATGCTCTCCTACCAAGACAAGGTGGGTGCCTTCTATAAGGACAATGCAAG

AGCTAATTCATCTAGGCTGTCCTTAGTGACAGAAGACCAAGGGGGCAGGAGACCACCCTATTTGCTGTTT

GTCCTGCTCTTCCTACTGGTTGGAATCATGGCCTTGCTCGCTATTACTGGAGTTCGATTTCACCAAGTAT

CAACTAGCAATATGGAATTTAGCAGATTGCTGAAAGAGGATATGGAGAAATCAGAGGCCGTACATCACCA

AGTCATAGATGTCTTGACACCACTCTTCAAAATTATTGGAGATGAGATTGGGTTACGGTTGCCACAAAAA

CTGAACGAGATCAAACAATTTATCCTTCAAAAGACAAACTTCTTTAATCCGAACAGGGAGT

>PQ559107.1 Morbillivirus canis isolate W014A_Raccoon_NC fusion protein (F) and hemagglutinin (H) genes, partial cds

ATCGTCGCGAATTGTGCTTCTATACTCTGTAAGTGTTATAGCACAAGCACAATTATCAATCAGAGTCCTG

ATAAGTTGCTGACATTTATTGCCTCCGATACCTGTCCACTGGTTGAAATAGATGGTGTAACTATCCAGGT

TGGAGGGAGGCAGTACCCTGATGTGGTATACGAAAGCAGAGTTGCCTTAGGCCCTGCTATATCACTTGAG

AGGTTAGATGTAGGGACAAATTTAGGGAACGCCCTTAAGAAACTGGATGATGCCAAAGTACTGATAGACT

CCTCTAACCAGATCCTTGAGACGGTTAGGCGCTCTTCCCTTAATTTTGGCAGTCTCCTCAGCGTTCCCAT

ATTAATCTGTACAGCTCTGGCTTTGTTGTTGCTAATCTACTGCTGTAAGAGACGCTACCAACAGACACTC

AGGCAGAACCCTAAGGTCGATCCTACATTTAAACCTGATTTGACTGGAACTTCGAAATCCTATGTAAGAT

CACTCTGAAGCACTCTGGTCACACGTGTTACCCGATGGTCAGGCTTGAAATCTATAAATCCCGCCCAATT

TCCCTCAAAGGCTATCAAACTGCAACAAATTGCGGAGAGGACGAACTACGATTCCCGTAATTAAAGAAAA

CTTAGGGCTCAGGTAGTCCAACAATGCTCTCCTACCGAGACAAGGTGGGTGCCTTCTATAAGGACAATGC

TAGAGCTAATTTATCCAAGCTGTCCTTAGTGACAGAAGAGCAAGGGGGCAGGAGACCACCCTATTTACTG

TTTGTCCTTCTCATCCTACTGGTTGGAATCATGGCCTTGCTTGCTATCACAGGAGTTCGATTTCACCAAG

TATCAACTAGCAATATGGAATTTAGCAGATTGCTGAAAGAGGATCTGGAGAAATCAGAGGCCGTACATCA

CCAAGTCATAGATGTCTTGACGCCGCTCTTCAAAATTATTGGAGATGAGATTGGGTTACGGTTGCCACAA

AAACTAAACGAGATCAAACAATTTATCCTTCAAAAAACAAACTTCTTCAATCCGAACAGGGAATTC

>PQ559108.1 Morbillivirus canis isolate W015A_Raccoon_NC fusion protein (F) and hemagglutinin (H) genes, partial cds

ATCGTCGCGAATTGTGCTTCTATACTCTGTAAGTGTTATAGCACAAGCACAATTATCAATCAGAGTCCTG

ATAAGTTGCTGACATTTATTGCCTCTGATACCTGTCCACTGGTTGAAATAGATGGTGTAACTATCCAGGT

TGGAGGGAGGCAGTACCCTGATATGGTATACGAAAGCAGAGTTGCCTTAGGCCCTGCTATATCGCTTGAG

AGGTTAGATGTAGGGACAAATTTGGGGAACGCCCTTAAGAAACTGGATGATGCCAAAGTACTGATAGACT

CCTCCAACCAGATTCTTGAGACGGTTAGGCGCTCTTCCCTTAATTTTGGCAGTCTCCTCAGCGTTCCCAT

ATTGATCTGTACAGCTCTGGCTTTGTTGTTGCTAATCTACTGCTGTAAGAGACGCTACCAACAGACACTC

AGGCAGAACCCTAAGGTCGATCCTACATTTAAACCTGATTTGACTGGAACTTCGAAATCCTATGTAAGAT

CACTCTGAAGCACTCTGGTCACACGTGTTACCCGATGGTCAGGCTTGAAATCTATGAATCCCGCCCAATT

TCCTTCAAAGGCTATCAAACTGCAACAAATTGTGGAGAGGACGAACTACGATTCCCGTAATTAAAGAAAA

CTTAGGGCTCAGGTAGTCCAACAATGCTCTCCTACCGAGACAAGGTGGGTGCCTTCTATAAGGACAATGC

TAGAGCTAATTTATCCAAGCTGTCCTTAGTGGCAGAAGAGCAAGGGGGCAGGAGACCACCCTATTTGCTG

TTTGTCCTTCTCATCCTACTGGTTGGAATCATGGCCTTGCTTGCTATCACTGGAGTCCGATTTCACCAAG

TATCAACTAGCAATATGGAATTTAGCAGATTGCTGAAAGAGGATCTGGAGAAATCAGAGGCCGTACATCA

CCAAGTCATAGATGTCTTGACGCCGCTCTTCAAAATTATTGGAGATGAGATTGGGTTACAGTTGCCACAA

AAACTAAACGAGATCAAACAATTTATCCTTCAAAAGACAAAATTCTTCAATCCGAACAGGGAATTC

>PQ559109.1 Morbillivirus canis isolate W016A_Raccoon_NC fusion protein (F) and hemagglutinin (H) genes, partial cds

ATCGTCGCGAATTGTGCTTCTATACTCTGTAAGTGTTATAGCACAAGCACAATTATCAATCAGAGTCCTG

ATAAGTTGCTGACATTTATTGCATCCGATACCTGTCCACTGGTTGAAATAGATGGTGTAACTATCCAGGT

TGGAGGGAGGCAGTACCCTGATGTGGTATACGAAAGCAGAGTTGCCTTAGGCCCTGCTATATCACTTGAG

AGGTTAGATGTAGGGACAAATTTAGGGAACGCCCTTAAGAAACTGGATGATGCCAAAGTACTGATAGACT

CCTCTAACCAGATCCTTGAGACGGTTAGGCGCTCTTCCCTTAATTTTGGCAGTCTCCTCAGCGTTCCCAT

ATTAATCTGTACAGCTCTGGCTTTGTTGTTGCTAATCTACTGCTGTAAGAAACGCTACCAACAGACACTC

AGGCAGAACCCTAAGGTCGATCCTACATTTAAACCTGATTTGACTGGAACTTCGAAATCCTATGTAAGAT

CACTCTGAAGCACTCTGGTCACACGTGTTACCCGATGGTCAGGCTTGAAATCTATAAATCCCGCCCAATT

TCCCTCAAAGGCTATCAAACTGCAACAAATTGCGGAGAGGACGAACTACGATTCCCGTAATTAAAGAAAA

CTTAGGGCTCAGGTAGTCCAACAATGCTCTCCTACCGAGACAAAGTGGGTGCCTTCTATAAGGACAATGC

TAGAGCTAATTTATCCAAGCTGTCCTTAGTGACAGAAGAGCAAGGGGGCAGGAGACCACCCTATTTACTG

TTTGTCCTTCTCATCCTACTGGTTGGAATCATGGCCTTGCTTGCTATCACAGGAGTTCGATTTCACCAAG

TATCAACTAGCAATATGGAATTTAGCAGATTGCTGAAAGAGGATCTGGAGAAATCAGAGGCCGTACATCA

CCAAGTCATAGATGTCTTGACGCCGCTCTTCAAAATCATTGGAGATGAGATTGGGTTACGGTTGCCACAA

AAACTAAACGAGATCAAACAATTTATCCTTCAAAAAACAAACTTCTTCAATCCGAACAGAGAATTC

>PQ559110.1 Morbillivirus canis isolate W050A_Raccoon_NC fusion protein (F) and hemagglutinin (H) genes, partial cds

AGTCCTGATAAGTTGCTGACATTTATTGCCTCCNATACCTGTCCACTGGTTGAAATAGATGGTGTAACTA

TCCAGGTTGGAGGGAGGCAGTACCCTGATATGGTATACGAAAGCAGAGTTGCCTTAGGCCCTGCTATATC

ACTTGAGAGGTTAGATGTAGGGACAAATTTAGGGAACGCCCTTAAGAAACTGGATGATGCCAAAGTACTG

ATAGACTCCTCTAACCAGATCCTTGAGACGGTTAGGCGCTCTTCCCTTAATTTTGGCAGTCTCCTCAGCA

TTCCTATATTAATCTGTACAGCTCTGGCTTTGTTGTTGCTAATCTACTGCTGTAAGAGACGCTACCAACA

GACACGCAGGCAGAACCCTAAGGTCGATCCTACATTTAAACCTGATTTGACTGGAACTTCGAAATCCTAT

GTAAGATCACTCTGAAGCACTCTGGTCACACGTGTTACCCGATGGTCAGGCTTGAAATCTATAAATCCCG

CCCAATTTCCTTCAAAGGCTATCATACTGCAACAAATTGTGGAGAGGACGAACTACGATTCCCGTAATTA

AAGAAAACTTAGGGCTCAGGTAGTCCAACAATGCTCTCCTACCGAGACAAGGTGGGTGCCTTCTATAAGG

ACAATGCTAGAGCTAATTTATCCAAGCTGTCATTAGTGACAGAAGAGCAAGGGGGCAGGAGACCACCCTA

TTTGCTGTTTGTCCTTCTCATCCTACTGGTTGGAATCATGGCCTTGCTTGCTATCACTGGAGTTCGATTT

CACCAAGTATCAACTAGCAATATGGAATTTAGCAGATTGCTGAAAGAGGATCTGGAGAAATCAGAGGCCG

TACAYCACCAAGTCATAGATGTCTTGACGCCGCTCTTCAAAATTATTGGAGATGAGATTGGGTTACGGTT

GCCACAAAAACTAAACGAGATYAAACAATTTATCCTTSAAAAGACAAACTTCTTCAATCCGAACAGGGA

>PQ559111.1 Morbillivirus canis isolate W062A_Raccoon_NC fusion protein (F) and hemagglutinin (H) genes, partial cds

ATCGTCGCGAATTGTGCTTCTATACTCTGTAAGTGTTATAGCACAAGCACAATTATCAATCAGAGTCCTG

ATAAGTTGCTGACATTTATTGCCTCCGATACCTGTCCACTGGTTGAAATAGATGGTGTAACTATCCAGGT

TGGAGGGAGGCAGTACCCTGATGTGGTATACGAAAGCAGAGTTGCCTTAGGCCCTGCTATATCACTTGAG

AGGTTAGATGTAGGGACAAATTTAGGGAACGCCCTTAAGAAACTGGATGATGCCAAAGTACTGATAGACT

CCTCTAACCAGATCCTTGAGACGGTTAGGCGCTCTTCCCTTAATTTTGGCAGTCTCCTCAGCGTTCCCAT

ATTAATCTGTACAGCTCTGGCTTTGTTGTTGCTAATCTACTGCTGTAAGAGACGCTACCAACAGACACTC

AGGCAGAACCCTAAGGTCGATCCTACATTTAAACCTGATTTGACTGGAACTTCGAAATCCTATGTAAGAT

CACTCTGAAGCACTCTGGTCACACGTGTTACCCGATGATCAGGCTTGAAATCTATAAATCTCGCCCAATT

TCCCTCAAAGGCTATCAAACTGCAACAAATTGCGGAGAGGACGAACTACGATTCCCGTAATTAAAGAAAA

CTTAGGGCTCAGGTAGTCCAACAATGCTCTCCTACAGAGACAAGGTGGGTGCCTTCTATAAGGACAATGC

TAGAGCTAATTTATCCAAGCTGTCCTTAGTGACAGAAGAGCAAGGGGGCAGGAGACCACCCTATTTACTG

TTTGTCCTTCTCATCCTACTGGTTGGAATCATGGCCTTGCTTGCTATCACAGGAGTTCGATTTCACCAAG

TATCAACTAGCAATATGGAATTTAGCAGATTGCTGAAAGAGGATCTGGAGAAATCAGAGGCCGTACATCA

CCAAGTCATAGATGTCTTGACGCCGCTCTTCAAAATTATTGGAGATGAGATTGGGTTACGGTTGCCACAA

AAACTAAACGAGATCAAACAATTTATCCTTCAAAAAACAAACTTCTTCAATCCGAACAGGGAATTC

>PQ559112.1 Morbillivirus canis isolate W089A_Raccoon_NC fusion protein (F) and hemagglutinin (H) genes, partial cds

ATCGTCGCGAATTGTGCTTCTATACTCTGTAAGTGTTATAGCACAAGCACAATTATCAATCAGAGTCCTG

ATAAGTTGCTGACATTTATTGCCTCCGATACCTGTCCACTGGTTGAAATAGATGGTGTAACTATCCAGGT

TGGAGGGAGGCAGTACCCTGATGTGGTATACGAAAGCAGAGTTGCCTTAGGCCCTGCTATATCACTTGAG

AGGTTAGATGTAGGGACAAATTTAGGGAACGCCCTTAAGAAACTGGATGATGCCAAAGTACTGATAGACT

CCTCTAACCAGATCCTTGAGACGGTTAGGCGCTCTTCCCTTAATTTTGGCAGTCTCCTCAGCGTTCCCAT

ATTAATCTGTACAGCTCTGGCTTTGTTGTTGCTAATCTACTGCTGTAAGAAACGCTACCAACAGACACTC

AGGCAGAACCCTAAGGTCGATCCTACATTTAAACCTGATTTGACTGGAACTTCGAAATCCTATGTAAGAT

CACTCTGAAGCACTCTGGTCACACGTGTTACCCGATGGTCAGGCTTGAAATCTATAAATCCCGCCCAATT

TCCCTCAAAGGCTATCAAACTGCAACAAATTGCGGAGAGGACGAACTACGATTCCCGTAATTAAAGAAAA

CTTAGGGCTCAGGTAGTCCAACAATGCTCTCCTACCGAGACAAAGTGGGTGCCTTCTATAAGGACAATGC

TAGAGCTAATTTATCCAAGCTGTCCTTAGTGACAGAAGAGCAAGGGGGCAGGAGACCACCCTATTTACTG

TTTGTCCTTCTCATCCTACTGGTTGGAATCATGGCCTTGCTTGCTATCACAGGAGTTCGATTTCACCAAG

TATCAACTAGCAATATGGAATTTAGCAGATTGCTGAAAGAGGATCTGGAGAAATCAGAGGCCGTACATCA

CCAAGTCATAGATGTCTTGACGCCGCTCTTCAAAATCATTGGAGATGAGATTGGGTTACGGTTGCCACAA

AAACTAAACGAGATCAAACAATTTATCCTTCAAAAAACAAACTTCTTCAATCCGAACAGAGAATTC

>PQ559113.1 Morbillivirus canis isolate W092A_Skunk_NC fusion protein (F) and hemagglutinin (H) genes, partial cds

ATCGTCGCGAATTGTGCTTCTATACTCTGTAAGTGTTATAGCACAAGCACAATTATCAATCAGAGTCCTG

ATAAGTTGCTGACATTTATTGCCTCCGATACCTGTCCACTGGTTGAAATAGATGGTGTAACTATCCAGGT

TGGAGGGAGGCAGTACCCTGATATGGTATACGAAAGCAGAGTTGCCTTAGGCCCTGCTATATCACTTGAG

AGGTTAGATGTAGGGACAAATTTAGGGAACGCCCTTAAGAAACTGGATGATGCCAAAGTACTGATAGACT

CCTCTAACCAGATCCTTGAGACGGTTAGGCGCTCTTCCCTTAATTTTGGCAGTCTCCTCAGCGTTCCTAT

ATTAATCTGTACAGCTCTGGCTTTGTTGTTGCTAATCTACTGCTGTAAGAGACGCTACCAACAGACACGC

AGGCAGAACCCTAAGGTCGATCCTACATTTAAACCTGATTTGACTGGAACTTCGAAATCCTATGTAAGAT

CACTCTGAAGCACTCTGGTCACACGTGTTACCCGATGGTCAGGCTTGAAATCTATAAATCCCGCCCAATT

TCCTTCAAAGGCTATCATACTGCAACAAATTGTGGAGAGGACGAACTACGATTCCCGTAATTAAAGAAAA

CTTAGGGCTCAGGTAGTCCAACAATGCTCTCCTACCGAGACAAGGTGGGTGCCTTCTATAAGGACAATGC

TAGAGCTAATTTATCCAAGCTGTCATTAGTGACAGAAGAGCAAGGGGGCAGGAGACCACCCTATTTGCTG

TTTGTCCTTCTCATCCTACTGGTTGGAATCATGGCCTTGCTTGCTATCACTGGAGTTCGATTTCACCAAG

TATCAACTAGCAATATGGAATTTAGCAGATTGCTGAAAGAGGATCTGGAGAAATCAGAGGCCGTACACCA

CCAAGTCATAGATGTCTTGACGCCGCTCTTCAAAATTATTGGAGATGAGATTTGGTTACGGTTGCCACAA

AAACTAAACGAGATCAAACAATTTATCCTTCAAAAGACAAACTTCTTCAATCCGAACAGGGAATTC

>PQ559114.1 Morbillivirus canis isolate W102A_Raccoon_MO fusion protein (F) and hemagglutinin (H) genes, partial cds

TTGTGCCTCTATACTATGTAAGTGTTATAGCACAGGCACAATTATCAATCAAAGTCCTGATAAATTGCTG

ACATTTATTGCCTCCGATACCTGCCCACTGGTTGAAATAGATGGTGTAACTATCCAGGTTGGAGGGAGGC

AATACCCTGATATGGTATACGAAAGCAAAGTCGCCTTGGGCCCTGCTATATCATTTGAGAGGTTAGATGT

AGGTACAAATTTAGGGAACGCTCTTAAGAAACTAGATGAGGCTAAGGTACTGATAGACTCCTCTAACCAG

ATCCTTGAAACGGTTAGGCGCTCTTCCTTTAATTTTGGCAGCCTTCTTAGTGTTCCCATATTAATCTGTA

CAGCCCTGGCTTTATTGTTGCTGATTTACTGCTGTAAAAGACGCTACCAACAGACACTCAAGCAGAATAC

TAAGGTCGATCCGACATTTAAACCTGATTTGACTGGAACTTCGAAATCCTATGTAAGATCACTCTGAGGC

ACTCTGCTCACACGTCTTACTCGATTGTCAGGCTTGAAATATATAAATTCCGCCCAATTTTCTTCAAAAG

CTATCAAACTGCAACAAATAGTGGAGAGGTCTGACTACGATTATCGTAATTAAAGAAAACTTAGGGCTCA

GGTAGTCCAACAATGCTCTCCTACCAAGACAAGGTGGGTGCCTTCTATAAGGACAATGCAAGAGCCAATT

CATCTAGGCTGTCCTTAGTGACGGAAGAACAAGGGGGCAGGAGACCACCCTATTTGCTGTTTGTCCTGCT

CATCCTACTGGTTGGAATCATGGCCTTGCTCGCTATTACTGGAGTTCGATTTCACCAAGTGTCAACTAGC

AATATGGAATTTAGCAGATTGCTGAAAGAGGATATGGAGAAATCAGAGGCCGTACATCACCAAGTCATAG

ATGTCTTGACACCACTCTTCAAAATTATTGGAGATGAGATTGGGTTACGGTTGCCACAAAAACTGAACGA

GATCAAACAATTTATCCTTCAAAAGACAAACTTCTTTAATCCGAACAGGGAGTTC

>PQ559115.1 Morbillivirus canis isolate W135A_Raccoon_NC fusion protein (F) and hemagglutinin (H) genes, partial cds

ATCGTCGTGAATTGTGCTTCTATACTCTGTAAGTGTTATAGCACAAGCACAATTATCAATCAGAGTCCTG

ATAAGTTGCTGACATTTATTGCCTCTGATACCTGTCCACTGGTTGAAATAGATGGTGTAACTATCCAGGT

TGGAGGGAGGCAGTACCCTGATATGGTATACGAAAGCAGAGTTGCCTTAGGCCCTGCTATATCGCTTGAG

AGGTTAGATGTAGGGACAAATTTAGGGAACGCCCTTAAGAAACTGGATGATGCCAAAGTACTGATAGACT

CCTCCAACCAGATTCTTGAGACGGTTAGGCGCTCTTCCCTTAATTTTGGCAGTCTCCTCAGCGTTCCCAT

ATTGATCTGTACAGCTCTGGCTTTGTTGTTGCTAATCTACTGCTGTAAGAGACGCTACCAACAGACACTC

AGGCAGAACCCTAAGGTCGATCCTACATTTAAACCTGATTTGACTGGAACTTCGAAATCCTATGTAAGAT

CACTCTGAAGCACTCTGGTCACACGTGTTACCCGATGGTCAGGCTTGAAATCTATGAATCCCGCCCAATT

TCCTTCAAAGGCTATCAAACTGCAACAAATTGTGGAGAGGACGAACTACGATTCCCGTAATTAAAGAAAA

CTTAGGGCTCAGGTAGTCCAACAATGCTCTCCTACCGAGACAAGGTGGGTGCCTTCTATAAGGACAATGC

TAGAGCTAATTTATCCAAGCTGTCCTTAGTGGCAGAAGAGCAAGGGGGCAGGAGACCACCCTATTTGCTG

TTTGTCCTTCTCATCCTACTGGTTGGAATCATGGCCTTGCTTGCTATCACTGGAGTCCGATTTCACCAAG

TATCAACTAGCAATATGGAATTTAGCAGATTGCTGAAAGAGGATCTGGAGAAATCAGAGGCCGTACATCA

CCAAGTCATAGATGTCTTGACGCCGCTCTTCAAAATTATTGGAGATGAGATTGGGTTACAGTTGCCACAA

AAACTAAACGAGATCAAACAATTTATCCTTCAAAAGACAAAATTCTTCANTCCGAACAGGGAATTC

>PQ559116.1 Morbillivirus canis isolate W144A_Raccoon_NC fusion protein (F) and hemagglutinin (H) genes, partial cds

TCAATCAGAGTCCTGATAAGTTGCTGACATTTATTGCCTCCGATACCTGTCCACTGGTTGAAATAGATGG

TGTAACTATCCAGGTTGGAGGGAGGCAATACCCTGATATGGTATACGAAAGCAGAGTTGCCTTAGGCCCT

GCTATATCACTTGAGAGGTTAGATGTAGGKACAAATTTAGGGAACGCCCTTAAGAAACTGGATGATGCYA

AAGTACTGATAGACTCCTCTAACCAGATCCTTGAGACGGTTAGGCGCTCTTCCYTTAATTTTGGCAGTCT

CCTCAGCGTTCCCATATTAATCTGTACAGCTCTGGCTTTRTTGTTGCTGATCTACTGCTGTAAGAGACGC

TACCAACAGACACTCAAGCAGAATCCTAAGGTCGATCCTACATTTAAACCTGATTTGACTGGAACTTCGA

AATCCTATGTAAGATCACTCTGAAGCACTCTGGTCACACGTGTTACCCGATGGTCAGGCTTGAAATATAT

AAATCCCGCCCAATTTCCCTCAAARGCTATCAAACTGCAACAAATAGTGGAGAGGACTAACTACGATTMY

CGTAATTAAAGAAAACTTAGGGCTCAGGTAGTCCAACAATGCTCTCCTACCGAGACAAGGTGGGTGCCTT

CTATAAGGACAATGCTAGAGCTAATTCATCCAAGCTGTCCTTAGTGACAGAAGAGCAAGGGGGCAGGAGA

CCACCCTATTTGCTGTTTGTCCTTCTCATCCTACTGGTTGGAATCATGGCCTTGCTTGCTATCACTGGAG

TTCGATTTCACCAAGTATCAACTAGCAATATGGAATTTAGCAGATTGCTGAAAGAGGATMTGGAGAAATC

AGAGGCCGTACATCACCAAGTCATAGATGTCTTGACGCCGCTCTTCAAAATTATTGGAGATGAGATTGGG

TTACGGTTGCCACAAAAACTRAACGAGATCAAACAATTTATCCTTCAAAAGACAAACTTCTTCAATCCGA

ACAGGGAATTC

>PQ559117.1 Morbillivirus canis isolate W153A_Raccoon_MO fusion protein (F) and hemagglutinin (H) genes, partial cds

TTGTGCCTCTATACTATGTAAGTGTTATAGCACAGGCACAATTATCAATCAAAGTCCTGATAAATTGCTG

ACATTTATTGCCTCCGATACCTGCCCACTGGTTGAAATAGATGGTGTAACTATCCAGGTTGGAGGGAGGC

AATACCCTGATATGGTATACGAAAGCAAAGTCGCCTTGGGCCCTGCTATATCACTTGAGAGGTTAGATGT

AGGTACAAATTTAGGGAACGCTCTTAAGAAACTAGATGAGGCTAAGGTACTGATAGACTCCTCTAACCAG

ATCCTTGAAACGGTTAGGCGCTCTTCCTTTAATTTTGGCAGCCTTCTTAGTGTTCCCATATTAATCTGTA

CAGCCCTGGCTTTATTGTTGCTGATTTACTGCTGTAAAAGACGCTACCAACAGACACTCAAGCAGAATAC

TAAGGTCGATCCGACATTTAAACCTGATTTGACTGGAACTTCGAAATCCTATGTAAGATCACTCTGAGGC

ACTCTGCTCACACGTCTTACTCGATTGTCAGGCTTGAAATATATAAATTCCGCCCAATTTTCTTCAAAAG

CTATCAAACTGCAACAAATAGTGGAGAGGTCTGACTACGATTATCGTAATTAAAGAAAACTTAGGGCTCA

GGCAGTCCAGCAATGCTCTCCTACCAAGACAAGGTGGGTGCCTTCTATAAGGACAATGCAAGAGCCAATT

CATCTAGGCTGTCCTTAGTGACGGAAGAACAAGGGGGCAGGAGACCACCCTATTTGCTGTTTGTCCTGCT

CATCCTACTGGTTGGAATCATGGCCTTGCTCGCTATTACTGGAGTTCGATTTCACCAAGTGTCAACTAGC

AATATGGAATTTAGCAGATTGCTGAAAGAGGATATGGAGAAATCAGAGGCCGTACATCACCAAGTCATAG

ATGTCTTGACACCACTCTTCAAAATTATTGGAGATGAGATTGGGTTACGGTTGCCACAAAAACTGAACGA

GATCAAACAATTTATCCTTCAAAAGACAAACTTCTTTAATCCGAACAGGGAGTTC

>PQ559118.1 Morbillivirus canis isolate W738A_Raccoon_NC fusion protein (F) and hemagglutinin (H) genes, partial cds

ATCGTCGCGAATTGTGCTTCTATACTCTGTAAGTGTTATAGCACAAGCACAATTATCAATCAGAGTCCTG

ATAAGTTGCTGACATTTATTGCCTCCGATACCTGTCCACTGGTTGAAATAGATGGTGTAACTATCCAGGT

TGGAGGGAGGCAGTACCCTGATATGGTATACGAAAGCAGAGTTGCCTTAGGCCCTGCTATATCACTTGAG

AGGTTAGATGTAGGGACAAATTTAGGGAACGCCCTCAAGAAACTGGATGATGCCAAAGTACTGATAGACT

CCTCTAACCAGATCCTTGAGACGGTTAGGCGCTCTTCCCTTAATTTTAGCAGTCTCCTCAGCGTTCCCAT

ATTAATCTGTACAGCTCTGGCTTTGTTGTTGTTAATCTACTGCTGTAAGAGACGCTACCAACAGACACTC

AGGCAGAACCCTAAGGTCGATCCTACATTTAAACCTGATTTGACTGGAACTTCGAAATCCTATGTAAGAT

CACTCTGAAGCGCTCTGGTCACAYGTGTTACCCGATGGTCAGGCTTGAAATCTATAAATCCCGCCCAATT

TCCTTCAAAGGCTATCAAACTGCAACAAATTGCGGAGAGGACGAACTACGATTCCCGTAATTAAAGAAAA

CTTAGGGCTCAGGTAGTCCAACAATGCTCTCCTACCGAGACAAGGTGGGTGCCTTCTATAAGGACAATGC

TAGAGCTAATTTATCCAAGCTGTCCTTAGTGACAGAAGAGCAAGGGGGCAGGAGACCACCCTATTTGCTG

TTTCTCCTCCTCATCCTACTGGTTGGAATCATGGCCTTGCTTGCTATCACTGGAGCCCGATTTCACCAAG

TATCAACTAGCAACATGGAATTTAGCAGATTGTTGAAAGAGGATCTGGAGAAATCAGAGGCCGTACATCA

CCAAGTCATAGATGTCTTGACGCCGCTCTTCAAAATTATTGGAGATGAGATTGGGTTACGGTTGCCACAA

AAACTAAACGAGATCAAACAATTTATCCTTCAAAAGACAAACTTCTTCAATCCGAACAGGGAATTC

>PQ559119.1 Morbillivirus canis isolate W791A_Raccoon_NC fusion protein (F) and hemagglutinin (H) genes, partial cds

TTGTGCCTCTATACTATGTAAGTGTTATAGCACAGGCACAATTATCAATCAAAGTCCTGATAAATTGCTG

ACATTTATTGCCTCCGATACCTGCCCACTGGTTGAAATAGATGGTGTAACTATCCAGGTTGGAGGGAGGC

AATACCCTGATATGGTATACGAAAGCAAAGTCGCCTTGGGCCCTGCTATATCACTTGAGAGGTTAGATGT

AGGTACAAATTTAGGGAACGCTCTTAAGAAACTAGATGAGGCTAAGGTACTGATAGACTCCTCTAACCAG

ATCCTTGAAACGGTTAGGCGCTCTTCCTTTAATTTTGGCAGCCTTCTTAGTGTTCCCATATTAATCTGTA

CAGCCCTGGCTTTATTGTTGCTGATTTACTGCTGTAAAAGACGCTACCAACAGACACTCAAGCAGAATAC

TAAGGTCGATCCGACATTTAAACCTGATTTGACTGGAACTTCGAAATCCTATGTAAGATCACTCTGAGGC

ACTCTGCTCACACGTCTTACTCGATTGTCAGGCTTGAAATATATAAATTCCGCCCAATTTTCTTCAAAAG

CTATCAAACTGCAACAAATAGTGGAGAGGTCTGACTACGATTATCGTAATTAAAGAAAACTTAGGGCTCA

GGCAGTCCAGCAATGCTCTCCTACCAAGACAAGGTGGGTGCCTTCTATAAGGACAATGCAAGAGCCAATT

CATCTAGGCTGTCCTTAGTGACGGAAGAACAAGGGGGCAGGAGACCACCCTATTTGCTGTTTGTCCTGCT

CATCCTACTGGTTGGAATCATGGCCTTGCTCGCTATTACTGGAGTTCGATTTCACCAAGTGTCAACTAGC

AATATGGAATTTAGCAGATTGCTGAAAGAGGATATGGAGAAATCAGAGGCCGTACATCACCAAGTCATAG

ATGTCTTGACACCACTCTTCAAAATTATTGGAGATGAGATTGGGTTACGGTTGCCACAAAAACTGAACGA

GATCAAACAATTTATCCTTCAAAAGACAAACTTCTTTAATCCGAACAGGGAGTTC

A.


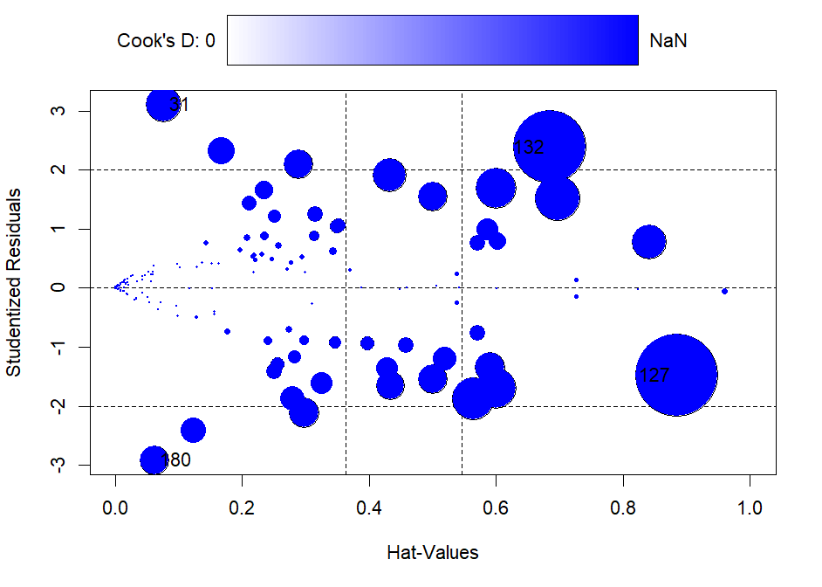


B.


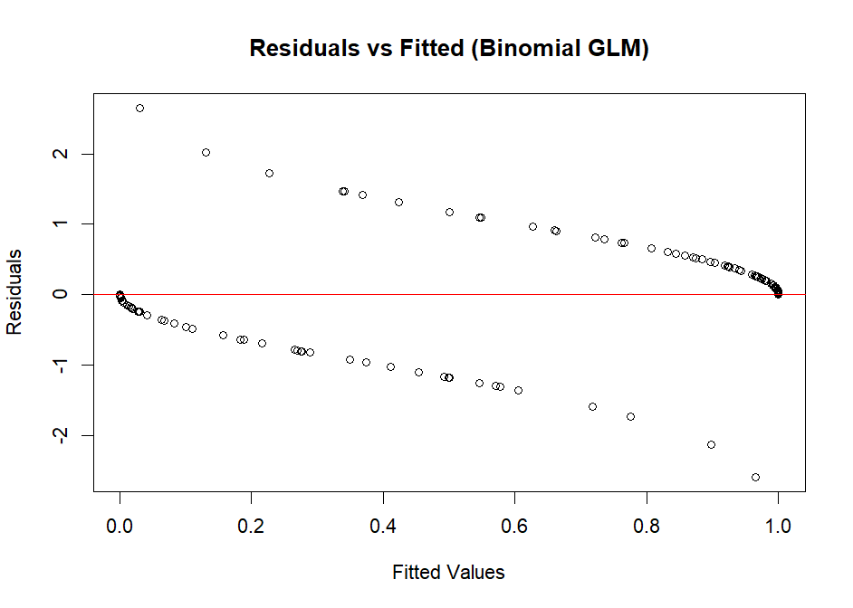


C.


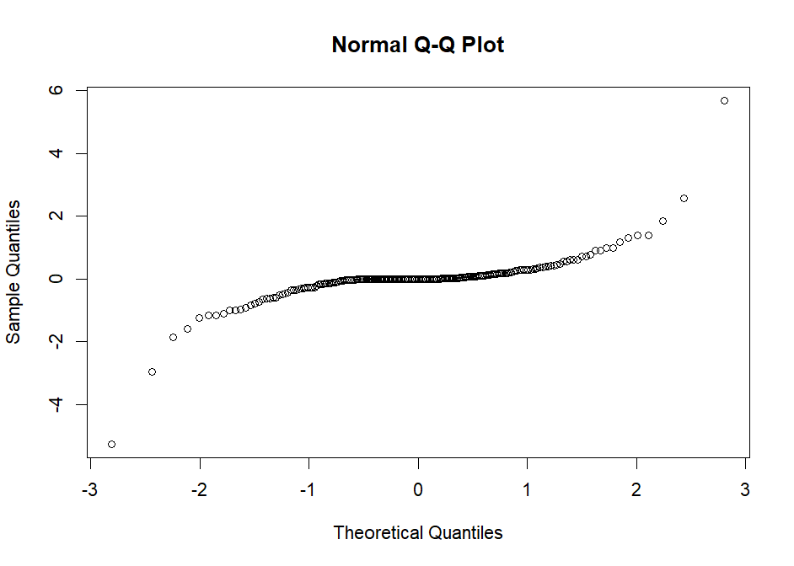


**Figure S1 (above/prior page). Model performance diagnostic plots for the best fit model. A. Influence plot (Hat values vs. standardized residuals), B. Residuals vs. Fitted plot, C. Normal Q-Q plot of Theoretical Quantiles vs. Sample Quantiles.**

**
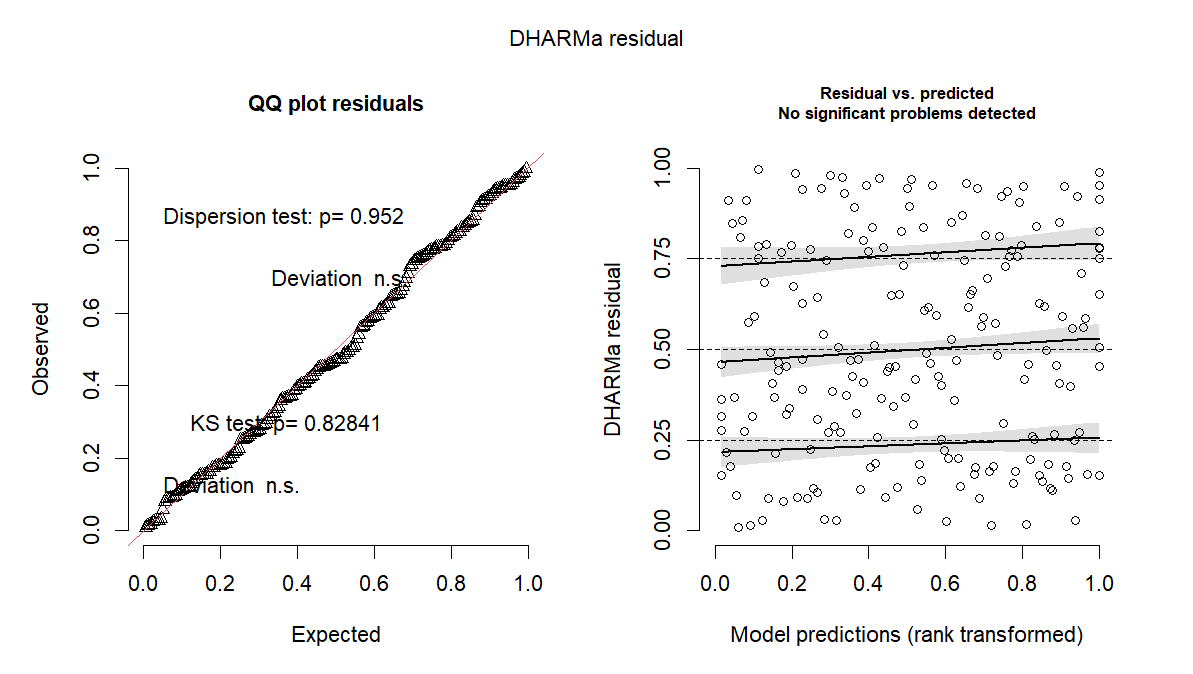
**

**Figure S2 (Above). DHARMa residual plots of residual diagnostics of the best fit model.** Left: QQ plot residuals (Expected vs. Observed), Right: Rank transformed model predictions versus simulated residuals show no significant model mis-specification problems. There is no over or underdispersion nor zero-inflation in the best fit model.


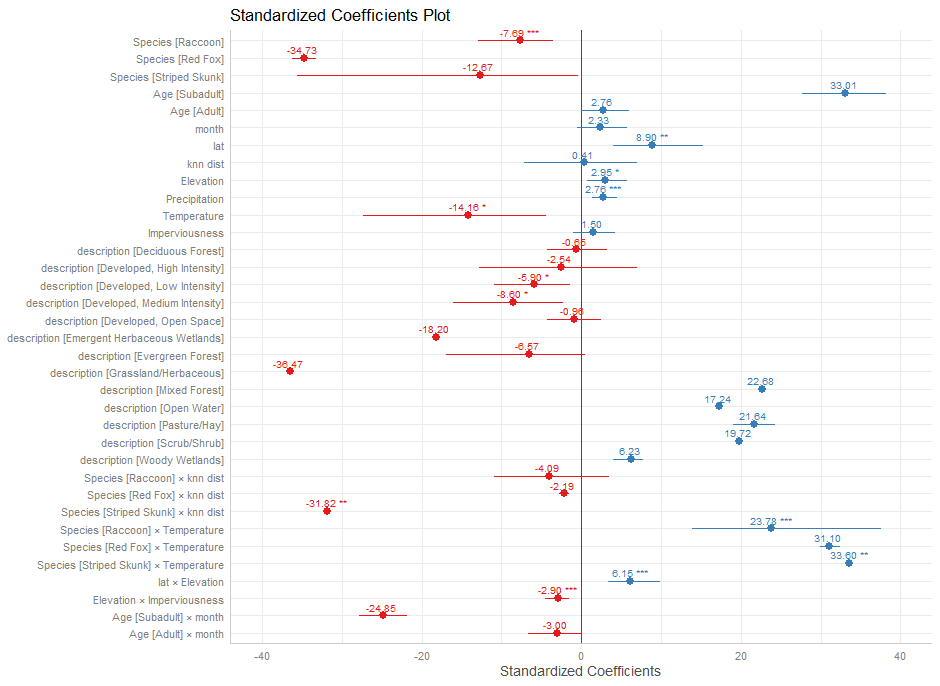


**Figure S3. Best fit model forest plot of standardized coefficients of predictors of canine distemper virus infection.** Stars indicate significant effects, * p<0.05, **p<0.005, ***p<0.0005.


**Supplementary figure 4A. Neighbor joining tree of the 32 CDV isolates collected during this study.**


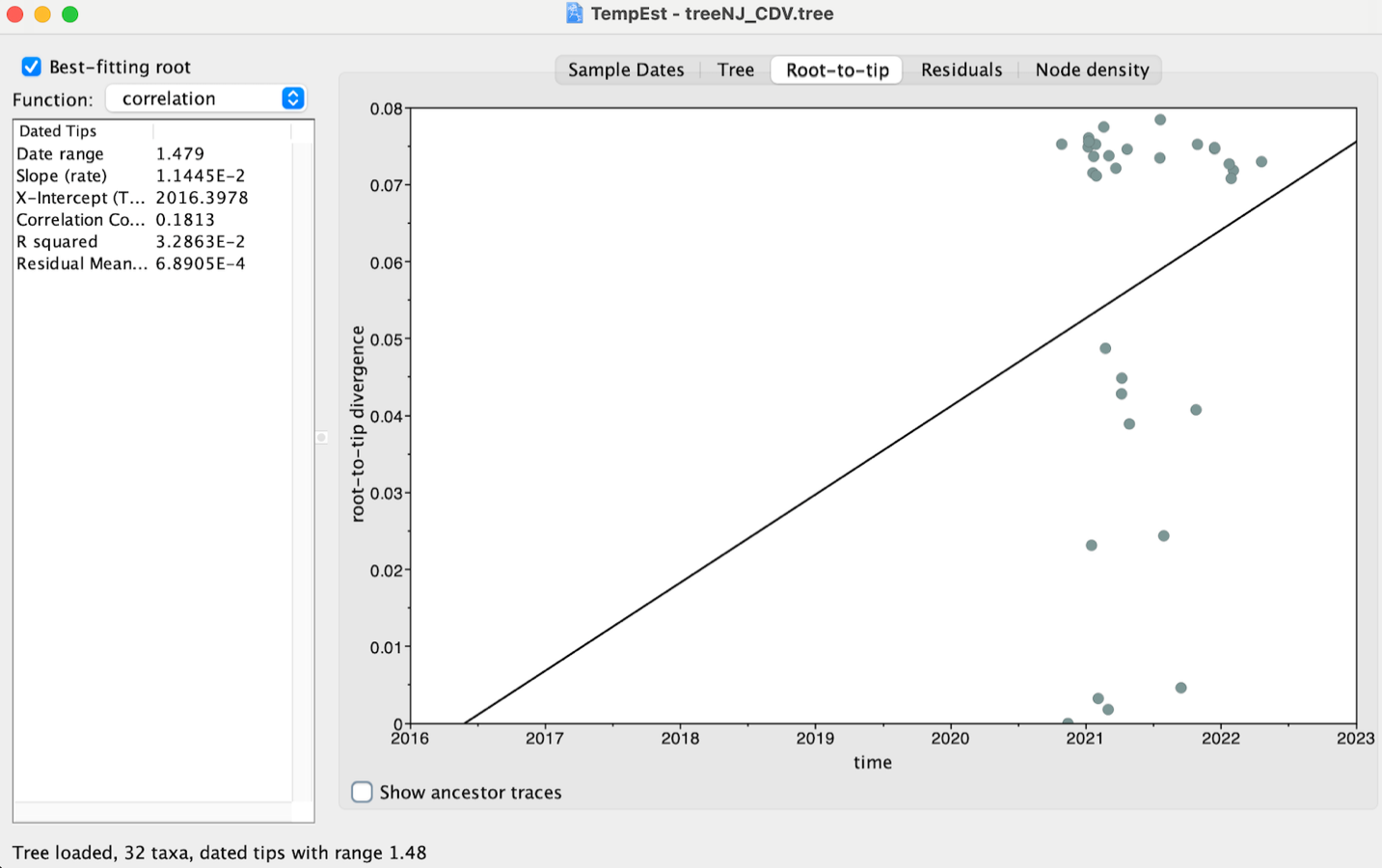


**Supplementary Figure 4B. Linear regression between root-to-tip distance and the sampling time for each CDV phylogenetic tree tip from Figure 1.**
